# Programmable Semi‐Interpenetrating Living Materials With Robust Stability for Versatile Bioremediation and Biotherapeutics

**DOI:** 10.1002/advs.202524320

**Published:** 2026-03-13

**Authors:** Zixian Bao, Shengfeng Yang, Dandan Hu, Jiezheng Liu, Bo Jiang, Xinyue Sui, Qingsheng Qi, Lai Li, Guang Zhao

**Affiliations:** ^1^ State Key Laboratory of Microbial Technology and Institute of Microbial Technology Shandong University Qingdao China; ^2^ Qingdao Central Hospital University of Health and Rehabilitation Sciences (Qingdao Central Hospital) Qingdao China; ^3^ CAS Key Laboratory of Biobased Materials Qingdao Institute of Bioenergy and Bioprocess Technology Chinese Academy of Sciences Qingdao China

**Keywords:** bioremediation, engineered living materials, hydroxybutyl chitosan, SpyCatcher/SpyTag, ulcerative colitis treatment

## Abstract

The practical application of engineered living materials (ELMs) is currently hindered by some critical challenges, such as streamlining fabrication processes and achieving long‐term stability. Here, a semi‐interpenetrating ELM was developed relying on thermosensitive self‐assembly of hydroxybutyl chitosan (HBC) and spontaneous covalent protein interactions. This semi‐interpenetrating network provided superior mechanical properties over HBC hydrogels. Furthermore, this material can be adapted for diverse scenarios based on engineered bacteria encapsulated, and its applications in biotherapy treatment and environmental remediation were validated. Compared to planktonic bacteria or enzymes, this ELM presented enhanced tolerance to harsh environments, including high temperatures, extreme pH values, high salinity, and digestive fluids, resulting in improved therapeutic efficacy with excellent biosafety in ulcerative colitis treatment and long‐term degradation of the pollutant paraoxon. In summary, our material offers advantages including simple preparation, excellent mechanical properties, high stability, customizability, and biosafety, laying a foundation for the application of ELMs.

## Graphical abstract

A semi‐interpenetrating engineered living material (sIHSELM), fabricated via thermosensitive hydroxybutyl chitosan and covalent protein self‐assembly, demonstrates exceptional mechanical properties and environmental stability. Shielding bacteria from harsh conditions, sIHSELM exhibits remarkable efficacy in ulcerative colitis treatment and pollutants degradation. This customizable and facile‐to‐prepare material offers excellent biosafety, establishing a foundation for the practical ELM applications.

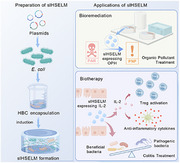

## 1 Introduction

Engineered living materials (ELMs) represent a burgeoning paradigm shift within the realms of materials science and engineering, characterized by the utilization of living components as fundamental building blocks and capitalizing on their inherent biological potential to fabricate “smart”, active, or multifunctional materials [1, 2, 3, 4]. By harnessing comprehensive synthetic biology techniques [5, 6, 7, 8], novel functionalities can be integrated into ELMs, paving the way for their exciting applications in building materials, wearable devices, biobrick fabrication, sensing, bioremediation, and biotherapeutic interventions [9, 10, 11, 12, 13, 14, 15]. The non‐living component of ELMs commonly takes the form of a hydrogel, which provides a suitable habitat for microbial cells and shields the bacteria from harsh environmental conditions, such as extreme temperatures, pH fluctuations, and mechanical stresses [16, 17, 18]. Several natural and synthetic hydrogels have been extensively employed in the fabrication of ELMs, such as chitosan, Pluronic F‐127, alginate, and high‐molecular polymers [3, 13, 16, 18, 19]. Although those ELMs exhibit exceptional functionalities, their environmental stability remains inadequately examined. Furthermore, the inherent limitations of these hydrogels, including complex fabrication processes, the necessity for chemical or photocrosslinking agents, potential adverse effects on bacteria, and suboptimal mechanical performance, hinder the broad practical deployment of ELMs.

Hydroxybutyl chitosan (HBC), a novel functional biomedical material, is a thermosensitive polymer synthesized through introducing hydroxybutyl side chains onto the active hydroxyl and amino groups of the chitosan backbone. This modification not only preserves the inherent biological activity of chitosan but also endows it with unique physicochemical properties and excellent biomedical properties [20, 21, 22]. For instance, the hydroxybutyl side chains effectively shield the hydrogen bonding sites both inside and outside the chitosan molecules, enabling HBC to exhibit excellent water solubility in physiological environments [21, 22]. Additionally, this modification strategy allows HBC to better mimic the extracellular matrix microenvironment and significantly enhances its biocompatibility [20]. The most prominent characteristic of HBC is its reversible temperature‐responsive phase transition behavior. At temperatures below the lower critical solution temperature (LCST), the polymer chains form a stable hydration layer with water molecules through hydrogen bonding, resulting in a homogeneous solution state. Conversely, when the temperature exceeds the LCST, the hydrophobic interactions become dominant, leading to the disruption of the hydration layer, dehydration of the polymer chains, and intermolecular entanglement, which completes the phase transition from solution to hydrogel within 1 min [22, 23]. Additionally, compared with traditional hydrogel systems, a key advantage of HBC lies in the simplicity and mildness of its preparation processes. HBC does not necessitate the use of acidic or alkaline agents for dissolution, thereby avoiding irreversible damage to biomolecules caused by the extreme pH conditions. The gelation mechanism is based on physical crosslinking (hydrophobic interaction and hydrogen bonding), which completely eliminates the need for chemical crosslinking agents. Consequently, these characteristics render HBC an attractive candidate for the fabrication of ELMs, as it preserves the metabolic activity of encapsulated cells, maintains the biological activity of bioactive factors, and significantly simplifies the overall manufacturing process.

The formation of HBC hydrogels relies on thermally induced self‐assembly of hydrophobic HBC chains. However, this mechanism inherently limits their structural stability and mechanical robustness due to the reversible nature of non‐covalent interactions under physiological conditions [24], thus significantly restricting their potential application as a matrix for ELM fabrication. Fortunately, the development of semi‐interpenetrating polymer networks (semi‐IPNs) offers a promising solution to this issue. In semi‐IPNs, two or more components interlace through physical entanglement, thereby reinforcing the overall physical properties without requiring covalent crosslinking [19]. Proteins represent ideal candidates for constructing semi‐IPNs with HBC, as they can be produced using engineered bacterial strains and programmed with tailored functionalities. However, most protein‐based hydrogels necessitate a chemical crosslinking agent to form stable networks, which may introduce biocompatibility concerns. SpyCatcher and SpyTag, derived from the splitting of the second immunoglobulin‐like collagen adhesin domain (CnaB2) of the fibronectin‐binding protein (FbaB) from *Streptococcus pyogenes* [25, 26], provide a robust alternative. Under mild conditions, SpyCatcher and SpyTag can spontaneously form covalent isopeptide bonds, regardless of whether they are fused at the N‐ or C‐terminus [27, 28], ensuring stable functionality. Consequently, SpyCatcher‐SpyTag systems represent highly promising candidates for the construction of bioactive hydrogels and ELMs with tailored properties [19, 29, 30, 31, 32]. By integrating genetic circuit design with bacterial encapsulation techniques, engineered bacteria can produce and release Spy proteins into the encapsulation environment, where these proteins polymerize to form semi‐IPNs and enhance the mechanical strength of the material [19, 33]. Therefore, incorporating living bacteria into the HBC matrix and utilizing their products to form a semi‐IPN structure offers a promising strategy for addressing the inherent challenges of HBC, such as its suboptimal stability and mechanical robustness under certain conditions.

**FIGURE 1:**
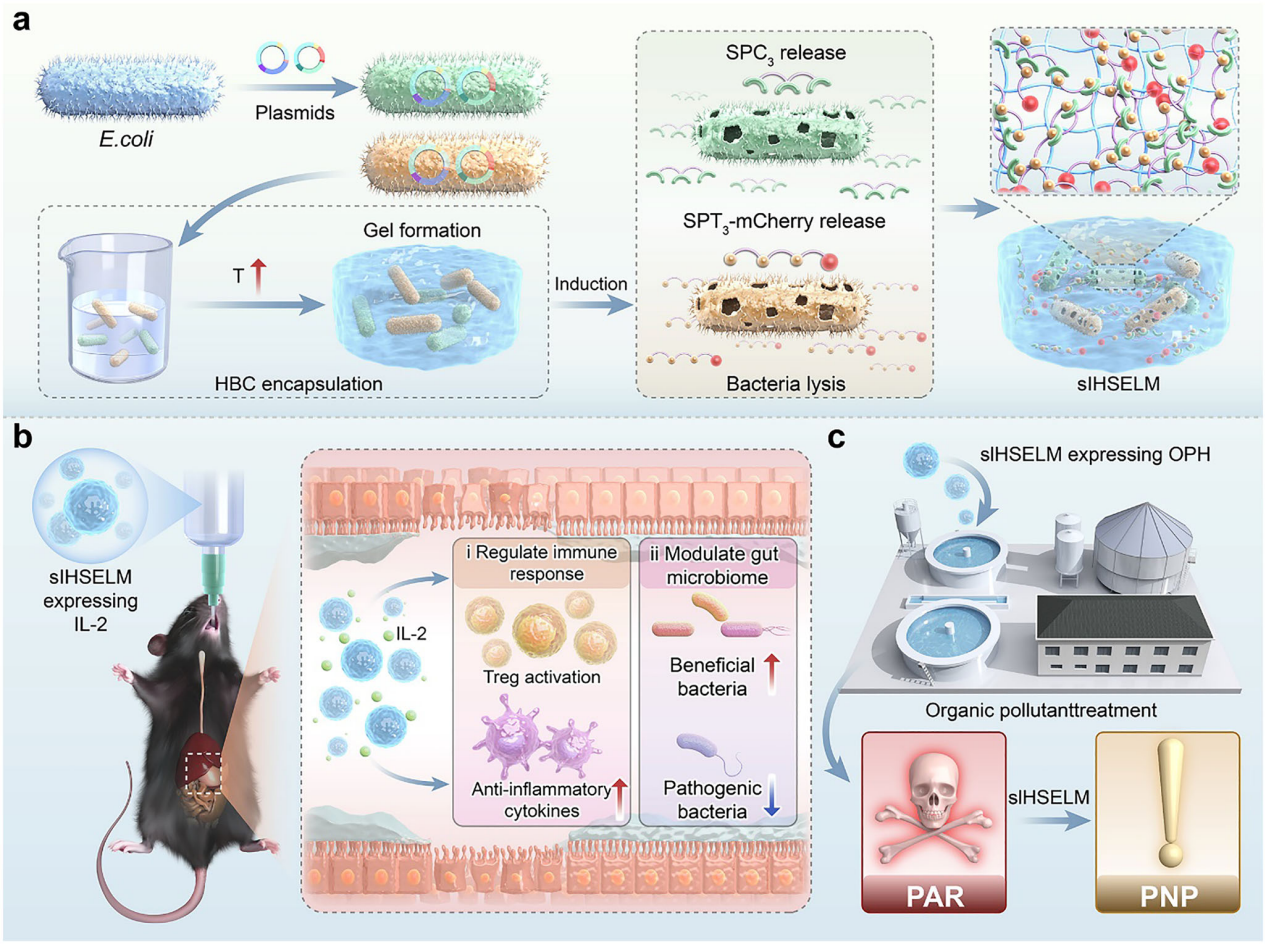
Fabricaiton of sIHSELM for biotherapeutics and bioremediation. (a) Schematic illustration of the fabrication process of sIHSELM. Bacteria were transformed with Spy proteins‐expressing plasmids and an inducible lysis plasmid, which were subsequently encapsulated using HBC solutions. Following heating and induction steps, Spy proteins were released into the HBC networks, where they underwent self‐polymerization to form the sIHSELM. (b) Therapeutic effect of sIHSELM expressing IL‐2 on ulcerative colitis. sIHSELM provided the protection to ECN, enhancing its resistance to the harsh conditions of the gastrointestinal tract environment. sIHSELM released IL‐2 to regulate the immune response and modulated gut microbiome, thereby achieving the treatment of ulcerative colitis. (c) Biodegradation capability of sIHSELM expressing organophosphate hydrolase (OPH) on organic pollutants. sIHSELM created a stable environment for OPH, allowing for long‐term maintenance of its enzymatic activity and the biodegradation of paraoxon (PAR) into *para*‐nitrophenol (PNP).

Herein, we proposed a facile and versatile construction strategy for the fabrication of stable ELMs with tailored functionalities, utilizing the unique properties of HBC. Bacteria expressed polymerized monomers (SpyCatcher and SpyTag tri‐block proteins) were encapsulated within the HBC hydrogel matrix through a straightforward procedure involving resuspension and mild heating. Subsequently, the semi‐interpenetrating HBC‐Spy engineered living material (sIHSELM) was synthesized via environmentally responsive stimuli (e.g., temperature shifts or chemical inducers), which triggered the controlled release of monomers and facilitated their subsequent polymerization within the hydrogel network (Figure 1). Notably, all fabrication steps were designed to be simple, biocompatible, cost‐effective, and environmentally sustainable, obviating the need for harsh conditions or toxic reagents. The resulting sIHSELM demonstrated exceptional stability across a broad pH range, effectively shielding the encapsulated bacteria from detrimental environments. Capitalizing on the programmability of the embedded bacteria, sIHSELM could be endowed with diverse functionalities, making it highly adaptable for various applications. Therefore, through the integration of HBC thermogelation and the bacteria‐mediated in situ covalent polymerization of Spy proteins, an ELM platform characterized by the straightforward preparation process, superior stability, and functional programmability can be fabricated. To this end, we verified the capacity of sIHSELM to efficiently alleviate intestinal inflammation and degrade environmental pollutants, respectively, underscoring its potential as a functional ELM that is highly suitable for applications in environmental remediation and biomedical therapeutics.

## 2 Results

### 2.1 Fabrication of sIHSELM

**FIGURE 2:**
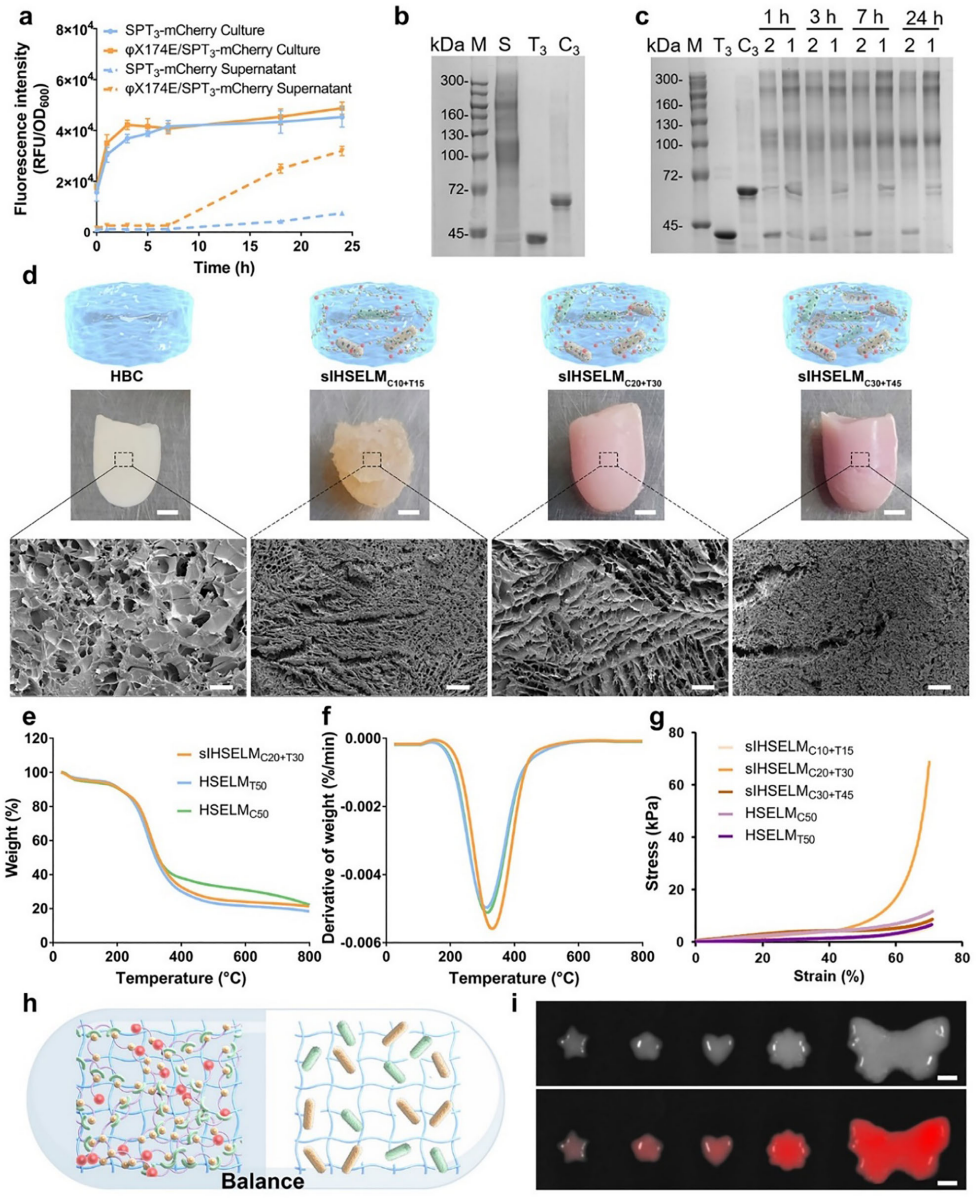
Fabrication and characterization of sIHSELM. (a) Fluorescence intensity of SPT_3_‐mCherry released from the culture medium or supernatant medium of the bacteria expressed with E protein (BL21‐φX174E/SPT_3_‐mCherry) or without E protein (BL21‐SPT_3_‐mCherry) (*n* = 3). (b) SDS‐PAGE analysis of the in vivo reaction between SPC_3_ (C_3_) and SPT_3_‐mCherry (T_3_) proteins released from the bacteria. S, purified supernatant proteins. (c) SDS‐PAGE analysis of in vitro reaction between purified SPC_3_ (C_3_) and SPT_3_‐mCherry (T_3_) proteins at a mole ratio of 1:2 (2) and 1:1 (1) within 24 h. (d) The representative photos and SEM images of HBC, sIHSELM_C10+T15_, sIHSELM_C20+T30_, and sIHSELM_C30+T45_. The scale bar in the hydrogel photos is 2 mm, and in the SEM images is 100 µm. (e, f) TGA (e) and DTG (f) curves of sIHSELM_C20+T30_, HSELM_C50,_ and HSELM_T50_. (g) Compressive stress‐strain curves of sIHSELM_C10+T15_, sIHSELM_C20+T30_, sIHSELM_C30+T45_, HSELM_C50_, and HSELM_T50_. (h) Schematic representation of the existence of a delicate balance between the disruptive effects of bacterial encapsulation and the stabilizing effects conferred by the semi‐IPNs. (i) The customized fabrication of sIHSELM with diverse conformations. The scale bar is 5 mm. Data are presented as mean values ± SD.

We delineated a meticulously engineered bacterial system designed to facilitate controlled cell lysis and subsequent protein release for the fabrication of advanced ELMs. The incorporated engineered bacteria expressed a lytic protein (E protein), which specifically interferes with bacterial cell wall synthesis, thereby inducing bacterial lysis and the concomitant release of intracellular proteins [34]. The expression of E protein was induced by either temperature or arabinose. To achieve temperature‐dependent lysis, we constructed a temperature‐sensitive lysis plasmid based on the pBV220 vector, which harbors the temperature‐sensitive λP_L_/P_R_‐CI^857^ fragment [35]. This plasmid enabled precise induction of bacterial lysis at 42°C. This precise induction mechanism allows for the decoupling of bacterial lysis from the expression of functional proteins. For validation purposes, *Escherichia coli* BL21(DE3) strains co‐transformed with the E protein‐expressing plasmid (pBV220‐φX174E) and an mCherry‐expressing plasmid (pACYCDuet‐mCherry) were initially cultured at 37°C for 3 h, followed by a temperature shift to 42°C and continued incubation for 24 h. Upon temperature‐induced lysis, a significant and early release of mCherry proteins into the culture medium was observed compared to the control group lacking the φX174E gene (Figure S2). Notably, the expression of the E protein indeed induced bacterial lysis, while viable bacteria were still present (Figure S3). Subsequently, Spy proteins were expressed using bacteria harboring the inducible lysis plasmid (BL21‐φX174E). To optimize their utility in ELMs fabrication, the Spy protein monomers (SpyCatcher or SpyTag) were genetically fused with hydrophilic elastin‐like polypeptides (ELPs) to generate three‐blocked Spy proteins (SPC_3_ or SPT_3_‐mCherry) [29], wherein mCherry was fused to the C‐terminus of SPT_3_ as a model functional protein. To confirm the successful expression of SPC_3_ and SPT_3_‐mCherry, Ni‐NTA affinity chromatography purification and SDS‐PAGE analysis were performed. As illustrated in Figure S4, distinct bands corresponding to SPC_3_ and SPT_3_‐mCherry were clearly observed, thereby verifying the successful construction of engineered bacteria capable of expressing these recombinant proteins. The release of Spy proteins was then evaluated using BL21‐φX174E/SPT_3_‐mCherry, due to the inherent fluorescence of mCherry for facile detection. As depicted in Figure 2a, a significantly higher fluorescent signal was detected in the supernatant of BL21‐φX174E/SPT_3_‐mCherry compared to the control group (BL21‐SPT_3_‐mCherry) within 24 h, indicating that the Spy proteins were efficiently released into the extracellular environment via E protein‐induced bacterial lysis. We then co‐cultured bacteria expressing SPC_3_ and SPT_3_‐mCherry (BL21‐φX174E/SPC_3_ and BL21‐φX174E/SPT_3_‐mCherry) at 42°C to induce simultaneous lysis and protein release. Following incubation, the culture supernatant was harvested and subjected to purification. SDS‐PAGE analysis revealed the formation of a series of high‐molecular‐weight protein complexes (72–300 kDa), as evidenced by the appearance of distinct bands at elevated molecular weights (Figure 2b). Similarly, when purified SPC_3_ and SPT_3_‐mCherry proteins were reacted in vitro, a comparable pattern of high‐molecular‐weight complexes emerged (Figure 2c). The similarity in band distribution between the purified supernatant proteins and the in vitro reaction groups strongly suggested that SPC_3_ and SPT_3_‐mCherry could be released into the culture medium and subsequently underwent spontaneous intermolecular reaction.

We investigated the lysis efficiency between the temperature‐induction system and the arabinose‐induction system (Figure S3). Following induction with either temperature (42°C) or arabinose (0.2 wt.%), no statistically significant differences were observed in the changes of OD_600 nm_ changes in both pBV220‐based groups. Conversely, in pBAD groups, the expression of E protein resulted in a significant decrease in OD_600 nm_ within 9 h (Figure S3c). Furthermore, a notable reduction in colony‐forming units (CFU) was observed in BL21(DE3) carrying the φX174E gene in both vectors (pBV220 and pBAD) when compared to the control group that harbored the φX174E gene (Figure S3a, b). These results demonstrated that the E protein could cause the lysis of bacteria. Notably, within 9 h, the lysis efficiency was higher in the pBAD‐φX174E/SPT_3_‐mCherry group than that in the pBV220‐φX174E/SPT_3_‐mCherry group.

HBC is a thermosensitive material that performs facile sol‐gel transition without using organic crosslinking agents, thereby preserving biocompatibility and simplifying processing protocols [21, 22]. We utilized the thermoresponsive properties of HBC to encapsulate the bacteria expressing functional Spy proteins under low temperature, followed by gelation induced by controlled heating. Concurrently, Spy proteins were released from lysed bacteria and self‐polymerized to form a semi‐IPNs with HBC chains. This approach culminated in the development of a versatile sIHSELM platform with tailored functionalities. HBC, exhibiting a gelation temperature of 18.1°C and a gelation time of 15 s, was employed in this study (Figure S5). To construct living sIHSELMs, defined CFUs of BL21‐φX174E/SPC_3_ and BL21‐φX174E/SPT_3_‐mCherry were mixed in a predetermined ratio (1:1.5), as the optimal reaction ratio between SPC_3_ and SPT_3_‐mCherry proteins was confirmed to be between 1:1 and 1:2 (SPC_3_:SPT_3_‐mCherry) (Figure 2c). The bacterial suspensions were then centrifuged to harvest the pellets, followed by resuspension in HBC solution (5 wt.%). The resulting composite materials were designated as sIHSELM_C10+T15_, sIHSELM_C20+T30_, sIHSELM_C30+T45_, where the subscripts denote the specific Spy protein type (C for SpyCatcher, T for SpyTag‐mCherry) and the corresponding CFUs of encapsulated bacteria used in each formulation. As a control, individual bacterial strains (BL21‐φX174E/SPC_3_ or BL21‐φX174E/SPT_3_‐mCherry) were processed identically to generate HBC‐Spy engineered living materials (HSELMs). These control materials were labeled as HSELM_C25_, HSELM_T25_, HSELM_C50_, HSELM_T50_, HSELM_C75_, HSELM_T75_, respectively.

The properties of sIHSELM were found to be significantly influenced by the encapsulation load of bacteria. The morphology of sIHSELM remained intact in the sIHSELM_C20+T30_ group, while large gullies were shown on the surface of other groups (sIHSELM_C10+T15_, sIHSELM_C30+T45_, HSELM_C50,_ and HSELM_T50_), resulting in an uneven colloid structure (Figure 2d and Figure S6). Scanning electron microscopy (SEM) analysis revealed that the sIHSELM_C20+T30_ group maintained regular and porous microstructures similar to those of pure HBC (pore size 80.2 ± 30 µm) (Figure 2d), with a notable reduction in pore size (58.8 ± 24.4 µm) due to the formation of semi‐IPN. In contrast, the microstructures of sIHSELM_C10+T15_ (pore size 23.4 ± 6.5 µm), sIHSELM_C30+T45_ (pore size 13.2 ± 5.8 µm), HSELM_C50_ (pore size 20.9 ± 9.2 µm), and HSELM_T50_ (pore size 18.9 ± 7.7 µm) formulations exhibited significant collapse (Figure S7), indicating the disruption of their microstructural integrity. To evaluate the thermal stability of sIHSELM, thermogravimetric analysis (TGA) was conducted. The results revealed that the maximum thermal decomposition temperature (*T*_max_) of pure HBC was the highest among all tested samples. Notably, *T*_max_ exhibited a decreasing trend with increasing load of encapsulated bacteria (Figure S8, Table S1). This phenomenon might be attributable to the introduction of bacteria, which contained labile small molecules that disrupted the intermolecular interactions among HBC chains, thereby compromising thermal stability. Interestingly, the *T*_max_ of the sIHSELM_C20+T30_ formulation was higher than that of both HSELM_C50_ and HSELM_T50_ (Figure 2f). This enhanced thermal stability might be ascribed to the formation of semi‐IPNs, which partially compensated for the disruption of HBC intermolecular interactions caused by bacterial encapsulation. Compression testing demonstrated that only the sIHSELM_C20+T30_ formulation exhibited superior mechanical properties, with a compressive fracture stress of 67.4 kPa (Figure 2g). In contrast, all other groups, including sIHSELM_C10+T15_, sIHSELM_C30+T45_, HSELM_C50,_ and HSELM_T50_, displayed significantly lower compressive fracture stresses ranging from 6.1 to 10.9 kPa. Rheological analysis was performed to elucidate the performance disparities in sIHSELM arising from variations in bacterial encapsulation load. As illustrated in Figure S9, sIHSELM_C20+T30_ exhibited the highest storage modulus (G′) among all samples, irrespective of whether the measurement was conducted in strain sweep (10–15 kPa) or frequency sweep mode (7–15 kPa). In contrast, the pristine HBC showed a relatively low G′ of 0.4–0.6 kPa under both modes, due to its solely physically cross‐linked network. However, encapsulation of a lower amount of bacteria resulted in a reduction of G′ to 0.03–0.04 kPa in both modes (sIHSELM_C10+T15_). Conversely, encapsulation of a higher amount of bacteria enhanced the storage modulus of the material (sIHSELM_C30+T45_, with G′ values ranging from 0.9 to1.4 kPa in strain sweep mode and from 1.7 to 2.1 kPa in frequency sweep mode), although these values remained lower than those observed in the sIHSELM_C20+T30_ group. The findings are consistent with previous reports indicating that the formation of semi‐IPN can augment the mechanical strength [19]. These results collectively indicated that the encapsulation load of bacteria exerted a profound influence on the internal microstructure, thermal stability, and mechanical properties of sIHSELM. The encapsulation of bacteria exerted a disruptive effect on the formation of HBC networks, primarily attributable to steric hindrance and electrostatic interactions. Bacteria served as substantial physical barriers that interrupted the continuous self‐assembly of HBC chains. Additionally, the negatively charged bacterial surfaces potentially formed hydrogen bonds with HBC chains, thereby hindering HBC from participating in the intermolecular network formation and resulting in the network defects. When a low number of bacteria were encapsulated (sIHSELM_C10+T15_), the quantity of released Spy proteins was minimal, leading to the formation of a weak semi‐IPN. Consequently, this weak network struggled to counteract the disruptive effect induced by the bacteria. As a result, an irregular microstructure was formed (Figure 2d), accompanied by a notable reduction in the storage modulus (Figure S9). When a higher amount of bacteria was encapsulated (sIHSELM_C30+T45_), sufficient Spy proteins were released to facilitate the formation of semi‐IPN networks. This, in turn, enhanced the storage modulus compared to that of pristine HBC (Figure S9). Nevertheless, the substantial disruptive effect brought about by the large quantity of bacteria caused the storage modulus of sIHSELM_C30+T45_ to be lower than that of sIHSELM_C20+T30_. This intriguing observation suggested the existence of a delicate balance between the disruptive effects of bacterial encapsulation on HBC intermolecular interactions and the stabilizing effects conferred by the semi‐IPNs (Figure 2h). Given the superior performance of the sIHSELM_C20+T30_ formulation in terms of microstructural integrity, thermal stability, and mechanical strength, this specific composition was selected for all subsequent experiments unless otherwise specified.

The formation of semi‐IPN was further confirmed with a chitosanase degradation assay. The prepared sIHSELM and HBC bulk hydrogels were incubated with a chitosanase‐sodium acetate solution (pH 4.5, 1 U/mL) at 37°C for 4 h. Subsequently, the degradation rate and microstructure of the hydrogels were assessed. As depicted in Figure S10, the HBC hydrogel underwent nearly complete degradation, whereas the sIHSELM retained approximately 64% of its initial weight. Furthermore, in contrast to the microstructures of HBC and sIHSELM prior to degradation, the porous microstructures of sIHSELM were disrupted following treatment with chitosanase, resulting in the formation of larger, hollow pores at the periphery of the hydrogel. Conversely, within the network, a more compact and densely packed arrangement of micropores emerged. The porous structure in the interior regions of the hydrogel remained partially preserved. Notably, these microstructural features differed from those observed under acidic conditions (pH 2). When incubated in acidic conditions, the porous microstructures remained largely intact, albeit with slight compression (Figure S14). Therefore, the observed microstructural alterations following chitosanase treatment were attributed to the degradation of HBC within the sIHSELM, while the protein networks remained intact. These findings collectively suggest the formation of a semi‐IPN within the sIHSELM. The spatial distribution and viability of encapsulated cells within sIHSELM (without mCherry expression) were further investigated through staining with LIVE/DEAD BacLight Bacterial Viability Kits and subsequent observation using fluorescence microscopy. The results revealed a uniform distribution of bacteria throughout the sIHSELM matrix. The viability of encapsulated bacteria remained unaffected by the formation of semi‐IPN. The observed diffuse red fluorescence was attributed to the staining of the hydrogel, with minimal to no dead bacteria detected within 24 h (Figure S11). Afterward, we performed an analysis of the spatial distribution of released SPT_3_‐mCherry proteins by employing confocal 3D imaging. As illustrated in Figure S12, the Spy proteins exhibited a homogeneous distribution throughout the sIHSELM matrix. These findings indicated that both bacteria and Spy proteins could achieve a uniform distribution within the material, and confirmed the successful formation of the semi‐IPN within sIHSELM without exerting any adverse effects on the viability of encapsulated bacteria. Additionally, due to the thermoresponsive and 3D‐printable properties of HBC, sIHSELM could be fabricated into diverse desired forms (Figure 2i).

### 2.2 The Superior Environmental Stability of sIHSELM

**FIGURE 3:**
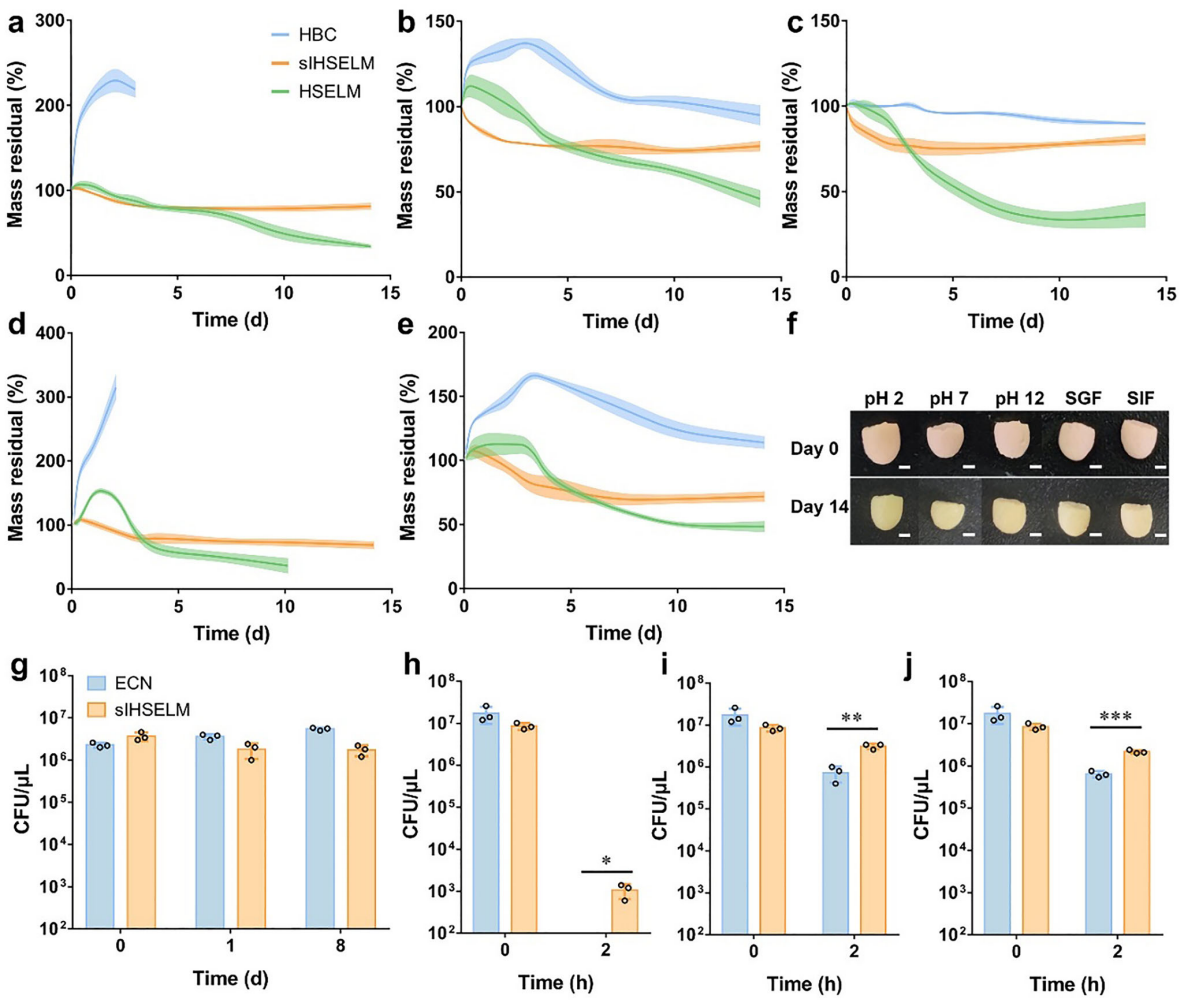
The stability evaluation of sIHSELM and its protection on bacteria. (a‐e) The stability of HBC, sIHSELM, and HSELM at a wide range of pH conditions, including pH 2 (a), pH 7 (b), and pH 12 (c), as well as in the SGF (d) and SIF (e). (f) The representative photos of sIHSELM before and after incubation in different conditions. The scale bar is 2 mm. (g‐j) Survival of bacteria without or with encapsulation within sIHSELM following storage at −80°C (g), and exposure to SGF (h), SIF (i), or bile salts (j). Data are presented as mean values ± SD (*n* = 3).

In light of the intended practical applications of the fabricated material, a comprehensive evaluation of its stability under diverse extreme environmental conditions is imperative. It is well‐documented that HBC hydrogel exhibits inherently limited stability [20], especially in acid or alkaline conditions [22], which substantially constrains its utility in real‐world scenarios. Previous studies have demonstrated that the incorporation of secondary components represents an effective strategy for enhancing hydrogel stability, as exemplified by the anchoring of diatom biosilica or the encapsulation of graphene oxide within the HBC matrix [36, 37, 38]. For sIHSELM, the encapsulation of bacteria expressing Spy proteins within the HBC matrix might influence its overall stability. To systematically investigate this hypothesis, the stability of pure HBC, sIHSELM, and HSELM was assessed under varying pH conditions, along with simulated gastric fluid (SGF) and simulated intestinal fluid (SIF). As illustrated in Figure 3a–e, the mass loss behavior of sIHSELM remained consistent across all tested conditions, exhibiting only slight mass reduction within the initial 3 days, followed by a plateau in weight change thereafter. Notably, the structural integrity of sIHSELM was preserved throughout the entire assessment period (Figure 3f). However, the pure HBC hydrogel demonstrated pronounced swelling behavior under acidic conditions (pH 2 and SGF) within the first 3 days, ultimately leading to complete dissolution in the solutions, indicating the poor stability of the HBC hydrogel in acidic conditions. Although the HBC hydrogel maintained its weight and shape for up to 14 days under physiological (pH 7) and alkaline (pH 12) conditions, its structure was compromised in SIF (Figure 3b, c, e, Figure S13). The formation of HBC hydrogel is dependent on the disruption of the hydration layer, dehydration of the polymer chains, and intermolecular entanglement. The hydrogen bonds formed within the entangled chains could be susceptible to disruption under acidic conditions, resulting in rehydration and the dissolution of HBC hydrogels. However, the incorporation of the semi‐IPN strengthened the stability of hydrogel networks, enabling the structure to be preserved even under acidic conditions. This was corroborated by the SEM images, which revealed that the structure of sIHSELM exhibited only slight compression when incubated under varying pH conditions (Figure S14), with the porous architecture remaining largely intact. For HSELMs, the mass loss ratio was consistently higher than that of sIHSELM groups incubated under identical conditions. This discrepancy may be attributed to the absence of specific interactions between Spy proteins in HSELMs, resulting in inferior structural stability. Furthermore, the thermal stability of sIHSELM was evaluated under different temperature conditions. The results revealed that even at elevated temperatures (> 42°C), sIHSELM retained over 60% of its initial weight while preserving its structural integrity (Figure S15). Collectively, these findings demonstrated that the encapsulation of bacteria expressing two distinct Spy proteins within the HBC matrix significantly enhanced the stability of the resulting composite material. Moreover, the presence of both Spy proteins appeared to be essential for maintaining structural integrity, as evidenced by the superior performance of sIHSELM compared to pure HBC and HSELMs. These results underscore the potential of sIHSELM as a robust platform for applications in harsh environmental conditions.

### 2.3 Protection Effect of Bacteria in sIHSELM In Vitro

Given the demonstrated exceptional stability of sIHSELM in both SGF and SIF, this material exhibits considerable potential as an oral delivery platform for therapeutic applications. To further validate this potential, we systematically investigated the survival of bacteria encapsulated within sIHSELM under SGF and SIF conditions. As a control, free bacteria (*E. coli* Nissle 1917, ECN) were incubated under identical conditions for comparative analysis. As illustrated in Figure 3h and Figure S16, free ECN exhibited rapid viability loss upon exposure to SGF, with complete mortality observed within 2 h. In contrast, the ECN encapsulated within sIHSELM maintained a viable cell count exceeding 10^3^ CFU µL^−1^ under the same conditions, underscoring the superior protective capacity of the HBC hydrogel matrix on ECN against the harsh acidic environment of the stomach. Subsequent evaluation of bacterial viability in SIF revealed that ECN encapsulated within sIHSELM also demonstrated significantly enhanced survival compared to free ECN (Figure 3i and Figure S16). Additionally, sIHSELM exhibited superior protection on encapsulated ECN when incubated with bile salts (Figure 3j and Figure S16). This finding suggested that the sIHSELM effectively shielded encapsulated bacteria from proteolytic degradation by trypsin, a key digestive enzyme abundant in the intestinal milieu.

Additionally, bacteria encapsulated within the sIHSELM demonstrated robust viability and successful resuscitation following cryopreservation at −80°C for a duration of 8 days. Throughout the entire freezing and thawing cycle, the viability of encapsulated bacteria remained statistically comparable to that of their pre‐frozen counterparts, as evidenced by quantitative CFU analysis (Figure 3g and Figure S17). Furthermore, no significant difference in viability was observed between the encapsulated bacteria and non‐encapsulated free ECN controls during the cryopreservation process (Figure 3g and Figure S17). These findings suggested that encapsulation within the sIHSELM did not adversely affect the viability of bacteria during cryogenic storage. From a practical perspective, this property enables long‐term storage of sIHSELM‐encapsulated bacteria at ‐80°C with reliable recovery of viable cells on demand, thereby offering significant advantages for applications requiring extended preservation periods, such as point‐of‐care intervention and controlled‐release therapeutic formulations.

### 2.4 Ulcerative Colitis Treatment of sIHSELM

Benefiting from the advancements in synthetic biology, proteins can be systematically engineered to execute distinct and specialized functions. By encapsulating bacteria engineered to express diverse proteins, sIHSELM can be imbued with multifaceted functionalities. Notably, sIHSELM demonstrated exceptional stability within simulated gastrointestinal tract environments, concurrently offering robust protection to the encapsulated bacteria (Figure 3d, e, h, i). These attributes collectively establish a solid foundation for its potential application in the oral delivery of therapeutic agents for disease treatment.

Inflammatory bowel disease (IBD) represents a chronic and recurrent inflammatory condition of the gastrointestinal tract, for which current medical interventions primarily involve the administration of aminosalicylates, antibiotics, corticosteroids, and immunosuppressants [39, 40, 41, 42]. However, these therapeutic strategies often fall short of achieving a complete cure, owing to underlying factors such as deregulated immune responses, intestinal mucosal damage, impaired intestinal barrier function, and dysbiosis of the gut microbiota within the GI tract [41, 43]. In recent years, probiotics have garnered significant attention as a promising therapy for IBD, attributable to their capacity to modulate the gut microbiome [41, 44, 45]. Nevertheless, the oral delivery of probiotics has been hampered by their low bioavailability, primarily due to the deleterious effects of gastric acid and bile salts [41, 46, 47, 48]. Consequently, there is an urgent need to devise effective protective strategies to overcome these challenges. Interleukin‐2 (IL‐2), an immunomodulatory cytokine with the ability to regulate immune responses, has been reported to alleviate inflammation and facilitate the repair of the colon epithelial barrier by modulating innate immune responses [49]. Building upon these insights, we encapsulated the ECN engineered to express IL‐2 within sIHSELM, and subsequently investigated the therapeutic potential of this system in the treatment of IBD. Despite the substitution of bacterial host strains and synthetic genetic circuits in the sIHSELM, efficient extracellular release of Spy proteins was still achievable through E protein‐mediated bacterial lysis (Figure S18).

The expression of IL‐2 is restricted within prokaryotic systems, primarily manifesting in the form of inclusion bodies. We attempted to express the SPT_3_‐IL‐2 fusion protein. However, the SDS‐PAGE results revealed the absence of detectable purified SPT_3_‐IL‐2 protein bands, thereby confirming its low expression efficiency when fused to the C‐terminus of SPT_3_ proteins (Figure S19). Nevertheless, the implementation of secretory expression strategies has the potential to improve the expression of IL‐2. Therefore, we opted to employ the SecB‐signal peptide (LamB) and fuse it at the N‐terminus of IL‐2 to realize the secretory expression [50]. Furthermore, an InfB solubility tag was fused at C‐terminus of IL‐2 to enhance its expression and solubility [51]. Subsequently, ECN strains expressing Spy proteins (ECN‐SPC_3_ and ECN‐SPT_3_‐mCherry) and secretory IL‐2 (ECN‐IL‐2) were co‐encapsulated within sIHSELM. Despite the introduction of a minor proportion of ECN‐IL‐2, the structural conformation of sIHSELM remained well‐preserved (Figure S20). The result indicated that the incorporation ratio of bacteria encapsulated within the sIHSELM system was not strictly fixed. This enables the concurrent introduction of a minor proportion of bacterial strains expressing alternative functional proteins, while maintaining the overall performance integrity of sIHSELM. This property holds significant importance for expanding the modular design of the sIHSELM system, particularly in scenarios where functional proteins exhibit low expression yields or are incompatible with fusion protein expression formats.

**FIGURE 4:**
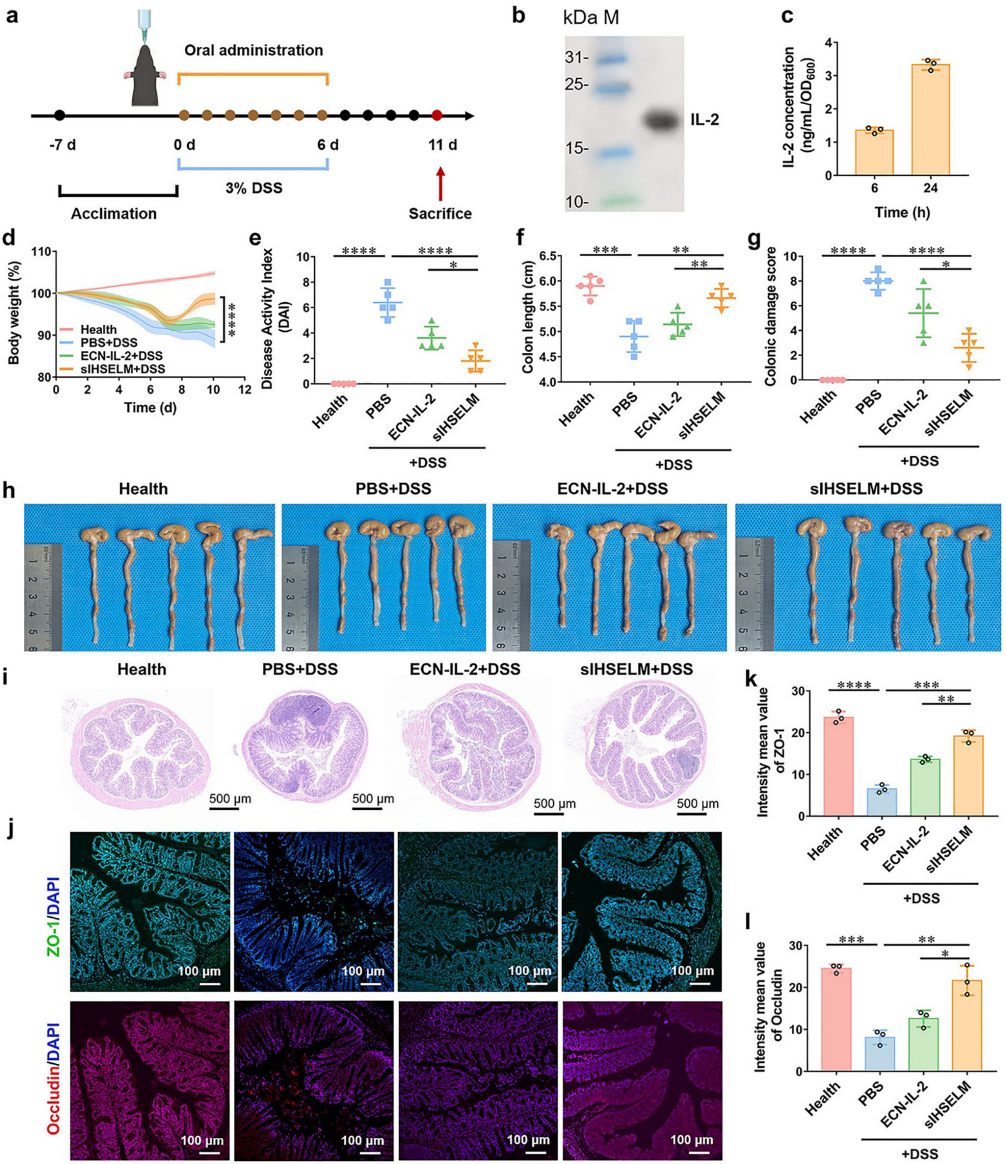
Therapeutic efficacy of sIHSELM against IBD. (a) Schematic diagram of the experimental procedure for the treatment of DSS‐induced IBD mice. Mice were acclimatized for 7 days. C57BL/6 mice were given drinking water containing 3% DSS for a period of 6 days. Meanwhile, the mice were subjected to daily oral gavage treatments over 6 consecutive days with PBS, ECN‐IL‐2 (1 × 10^9^ CFU), or sIHSELM (containing 1 × 10^9^ CFU of ECN‐IL‐2). The pattern was created with BioGDP.com [52]. (b) Western blot analysis of the secretory expression of IL‐2. (c) The secretion of IL‐2 from ECN‐IL‐2 within 24 h. (d) Changes in the mice's body weight with different treatments. (e) DAI of mice with different treatments on day 10. (f) The length and (h) images of the colon harvested from the mice with different treatments. (g) The colonic damage score of mice with different treatments. (i) Representative H&E staining images of the colon harvested from the mice with different treatments. The scale bar is 500 µm. (j) Representative immunofluorescence staining of tight junction‐associated protein, including ZO‐1 and Occludin. The scale bar is 100 µm. (k, l) Relative fluorescence intensity of ZO‐1 and Occludin in colon sections as shown in (j). Data are presented as mean values ± SD (*n* = 3 biologically independent samples for (c, j‐l), *n* = 5 biologically independent samples for (d–i)). ^*^ *p* < 0.05, ^**^ *p* < 0.01, ^***^ *p* < 0.001, ^****^ *p* < 0.0001.

To assess the expression and secretion of IL‐2 from ECN, Western blot analysis and enzyme‐linked immunosorbent assay (ELISA) were employed. As illustrated in Figure 4b, a distinct band corresponding to IL‐2 was observed in the supernatant fraction, confirming the successful expression and secretion of IL‐2 into the extracellular environment. Furthermore, the concentration of IL‐2 secreted into the supernatant was quantified using an ELISA kit. The results revealed a time‐dependent increase in IL‐2 secretion (Figure 4c), indicating that ECNs were capable of continuously producing and releasing IL‐2 into the extracellular environment over the course of the incubation period. Subsequently, we investigated the release behavior of IL‐2 from sIHSELM incubated in SIF. The quantification of released IL‐2 was performed by analyzing the centrifuged supernatant using the ELISA kit. The results demonstrated that sIHSELM was capable of sustaining the synthesis of IL‐2 within 48 h, with IL‐2 being released and diffusing from the sIHSELM into the surrounding SIF solution (Figure S21). The capacity of sIHSELM to sustainably synthesize and release IL‐2 is essential for its application in colitis treatment.

We then established an experimental model of IBD induced by dextran sodium sulfate (DSS) and assessed the therapeutic potential of sIHSELM in IBD‐afflicted mice (Figure 4a). C57BL/6 mice were administered a 3% DSS solution in their drinking water for a period of 6 days to induce acute colitis. Simultaneously, the mice were subjected to daily oral gavage treatments over 6 consecutive days with either PBS, ECN‐IL‐2 (1 × 10^9^ CFU), or sIHSELM (containing 1 × 10^9^ CFU of ECN‐IL‐2). Compared to the health group (PBS + water), all mice subjected to DSS treatment exhibited significant weight loss within the first 6 days (Figure 4d), confirming the successful establishment of the IBD model. In the sIHSELM‐treated group (sIHSELM + DSS), mice demonstrated a marked improvement in body weight beginning on day 7 post‐induction, with full recovery to normal levels achieved by day 10. In contrast, mice in the other treatment groups (PBS + DSS, ECN‐IL‐2 + DSS) failed to exhibit complete alleviation of weight loss within 10 days. The disease activity index (DAI) was evaluated on day 10, considering changes in body weight, fecal consistency, and presence of occult blood in stool (Table S2). The DAI scores of mice treated with sIHSELM were significantly lower than those observed in the other groups (PBS + DSS, ECN‐IL‐2 + DSS) (Figure 4e).

All the mice were euthanized on day 11, and their colons were harvested for further assessment of the therapeutic effect of sIHSELM. The colon lengths of mice in the sIHSELM‐treated group (sIHSELM + DSS) were significantly longer than those observed in the other IBD‐afflicted groups, and close to the lengths measured in the health group (Figure 4f, h). Subsequently, a histological evaluation of the colon was performed, focusing on epithelial damage and inflammatory cell infiltration as key indicators of disease severity (Table S3). As revealed in Figure 4g, i, colon samples derived from mice treated with sIHSELM (sIHSELM + DSS) displayed the lowest histopathological scores among all experimental groups. These samples were characterized by a regular fingerlike crypt structure, an intact epithelial layer, and a decreased percentage of inflammatory cells. These histological features in the sIHSELM‐treated group closely resembled those observed in healthy mice, suggesting that sIHSELM had the capacity to promote the repair of colon tissue damaged by colitis. We further investigated the impact of sIHSELM on the integrity of the colonic epithelial barrier, which is a critical determinant of disease progression in DSS‐induced acute IBD [53]. We examined the expression of key tight junction proteins, ZO‐1 and Occludin, which play pivotal roles in maintaining the structural and functional integrity of intestinal epithelial cells in DSS‐induced colitis [54]. Immunofluorescence staining revealed that, in the sIHSELM‐treated group (sIHSELM + DSS), the expression levels of both ZO‐1 and Occludin were significantly upregulated compared to those in the ECN‐IL‐2‐treated group (ECN‐IL‐2 + DSS) (Figure 4j–l).

**FIGURE 5:**
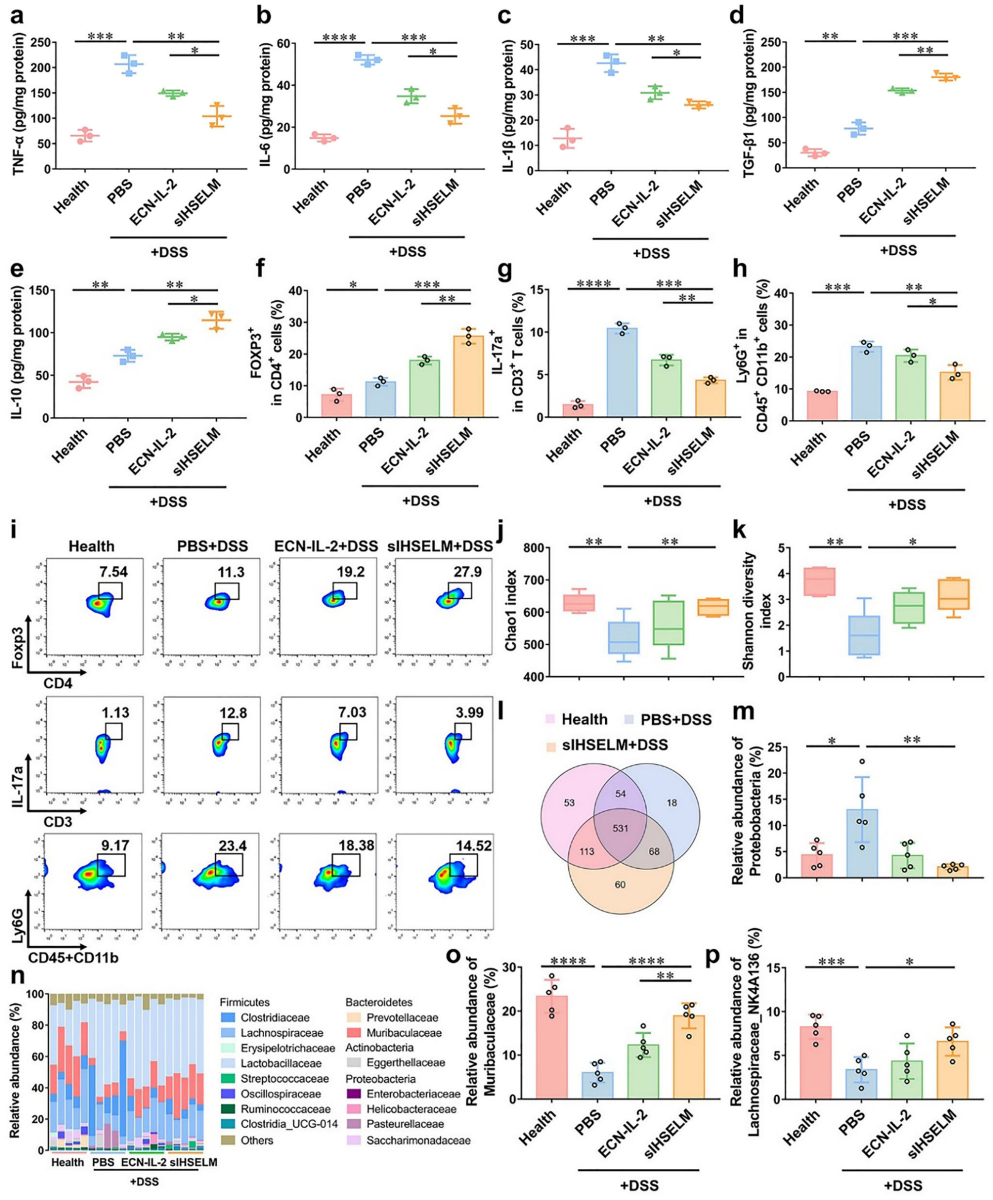
Mechanism of sIHSELM in ameliorating colitis and modulating the gut microbiome. (a–e) The levels of TNF‐α, IL‐6, IL‐1β, TGF‐β1, and IL‐10 in the colons were measured by ELISA. (f–i) Flow cytometric analysis (i) and statistical analysis of Foxp3^+^CD4^+^ (f), IL‐17a^+^CD3^+^ (g), and Ly6G^+^CD45^+^CD11b^+^ cells (h) in the colons harvested from the mice with different treatments. (j, k) Gut microbiome diversity analysis via the Chao1 index (j) and Shannon diversity index (k). (l) Venn diagram of the bacterial species in the colons harvested from the mice with different treatments. (n) Relative abundance of different families in the gut microbiota of mice with different treatments. (m, o, p) Relative abundances of Proteobacteria (m), Muribaculaceae (o), and Lachnospiraceae_NK4A136 (p). Data are presented as mean values ± SD (*n* = 3 biologically independent samples for (a‐i), *n* = 5 biologically independent samples for (j‐p)). ^*^ *p* < 0.05, ^**^ *p* < 0.01, ^***^ *p* < 0.001, ^****^ *p* < 0.0001.

The underlying mechanism of sIHSELM to ameliorate IBD was subsequently explored. To accurately assess the degree of inflammation in mice with acute IBD, colonic inflammatory markers were meticulously quantified using an ELISA assay. The results demonstrated that the levels of typical pro‐inflammatory cytokines, including tumor necrosis factor‐α (TNF‐α), interleukin‐6 (IL‐6), and interleukin‐1β (IL‐1β), were significantly attenuated in the sIHSELM‐treated group (sIHSELM + DSS) compared to other IBD‐afflicted groups (Figure 5a–c). Concurrently, the levels of anti‐inflammatory cytokines, including interleukin‐10 (IL‐10) and transforming growth factor‐β (TGF‐β), were correspondingly elevated in the sIHSELM treated group (sIHSELM + DSS) (Figure 5d,e). These findings collectively suggested the remarkable anti‐inflammatory effects exerted by sIHSELM. At a low concentration, IL‐2 has been shown to preferentially activate regulatory T cells (Tregs), a specialized subset of T cells that are essential for regulating immune responses [49, 55]. Additionally, IL‐17a‐producing T cells (Th17 cells) can modulate inflammatory responses by secreting IL‐17a, which subsequently activates and recruits neutrophils to sites of inflammation [56]. To further elucidate the immunomodulatory effects of sIHSELM, we employed flow cytometry to analyze the populations of Tregs, Th17 cells, and neutrophils in the colonic tissues of treated mice (Figures S22–S24). As depicted in Figure 5f–i, the proportion of Tregs (Foxp3^+^) was significantly increased in the IL‐2‐expressed groups (ECN‐IL‐2 + DSS and sIHSELM + DSS). In contrast, the populations of IL‐17a^+^CD3^+^ cells and neutrophils were significantly reduced compared to the untreated group (PBS + DSS). The results indicated that IL‐2 released from ECN could effectively modulate innate immune responses by promoting Treg activation and suppressing pro‐inflammatory pathways. These findings are consistent with previously reported results [49]. Notably, when compared to free ECN‐IL‐2, sIHSELM exhibited significantly enhanced immune modulation capabilities, which might be attributed to the fact that sIHSELM enhanced the survival of viable ECN‐IL‐2 delivered to the colon, thereby enabling the release of more IL‐2 to modulate immune response.

The gut microbiota has been widely recognized as a critical factor in both the pathogenesis of IBD and the maintenance of intestinal homeostasis [57, 58]. On day 10, fecal samples were collected from all mice and subjected to 16S rRNA sequencing to investigate the impact of sIHSELM on gut microbiota composition and diversity. Our analysis revealed that treatment with sIHSELM significantly enhanced gut microbiota diversity, as evidenced by notable increases in both the Chao1 and Shannon indices (Figure 5j,k). Furthermore, a Venn diagram analysis demonstrated that the sIHSELM‐treated group (sIHSELM + DSS) exhibited a greater number of unique and shared microbial taxa compared to other IBD‐afflicted groups (Figure 5l and Figure S25). At the phylum and family levels, taxonomic profiling revealed that sIHSELM intervention led to a marked enrichment of beneficial bacteria, including Muribaculaceae, Lachnospiraceae_NK4A136, Odoribacter (Figure 5n–p, and Figures S26 and S27) [59, 60]. Simultaneously, the relative abundance of Proteobacteria and Enterobacteriaceae, IBD‐promoting pathogenic bacteria [49, 60, 61], was significantly reduced following sIHSELM treatment (Figure 5m and Figure S27). These findings collectively suggested that sIHSELM intervention optimized gut microbiota composition by promoting the growth of beneficial commensal bacteria while suppressing pathogenic taxa. This modulation of the gut microbial ecosystem likely contributed to the enhanced therapeutic efficacy of sIHSELM against colitis, highlighting its potential as a novel strategy for IBD management.

### 2.5 Biosafety Evaluation of sIHSELM

**FIGURE 6:**
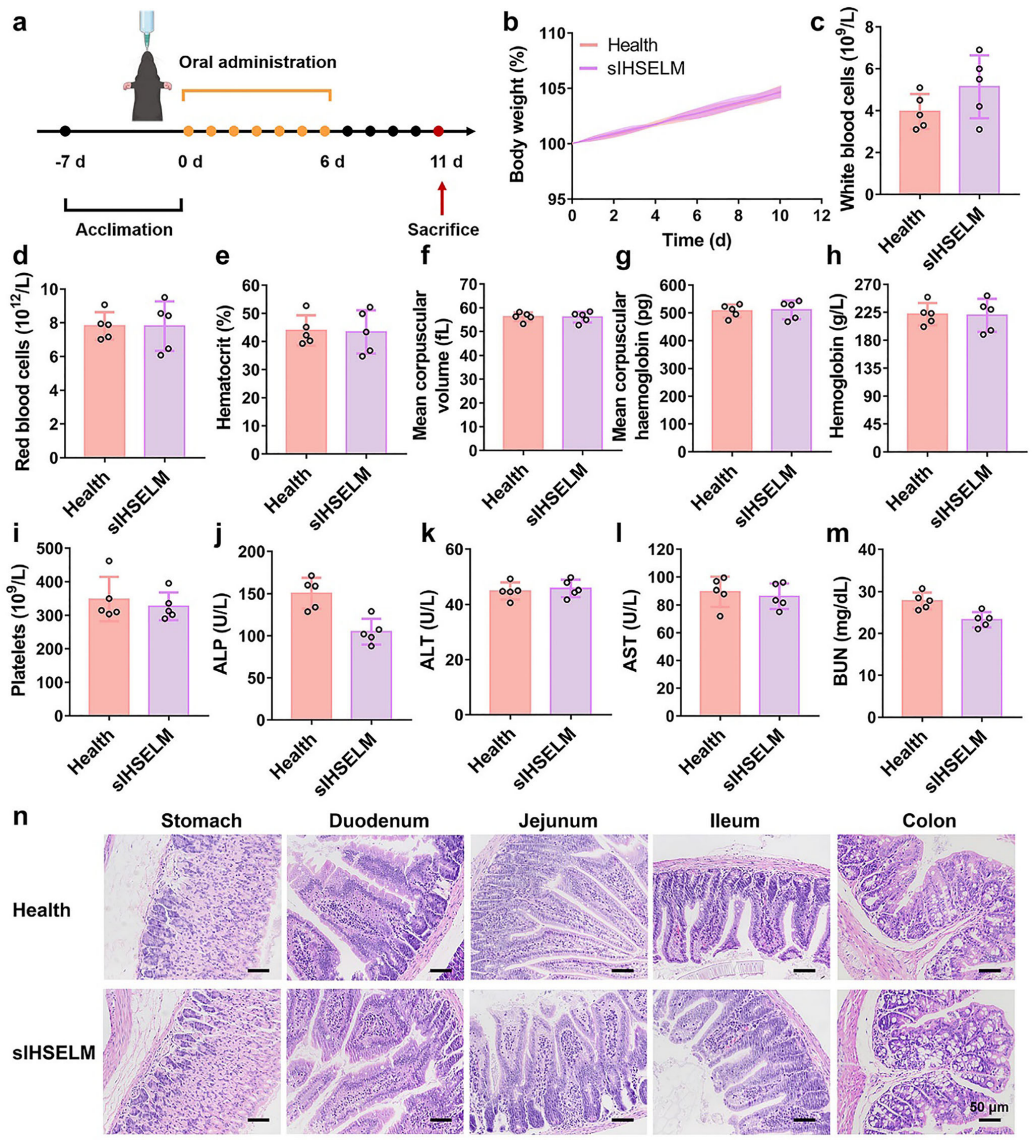
Biosafety of sIHSELM. (a) Schematic diagram of the experimental procedure for evaluating the biosafety of sIHSELM. Mice were acclimatized for 7 days. C57BL/6 mice were subjected to daily oral gavage treatments over 6 consecutive days with PBS or sIHSELM (containing 1 × 10^9^ CFU of ECN‐IL‐2). The pattern was created with BioGDP.com [52]. (b) Changes in the mice's body weight with different treatments. (c–m) Complete blood and serum biochemistry analysis of the mice with different treatments. ALP, alkaline phosphatase; ALT, alanine aminotransferase; AST, aspartate aminotransferase; BUN, blood urea nitrogen. (n) H&E staining sections of stomach, duodenum, jejunum, ileum, and colon harvested from the mice with different treatments. The scale bar is 50 µm. Data are presented as mean values ± SD (*n* = 5 biologically independent samples for (b–m)).

Given the promising therapeutic efficacy of sIHSELM against IBD, its biosafety was systematically evaluated through comprehensive in vivo investigations. Mice were administered sIHSELM (containing 1 × 10^9^ CFU of ECN‐IL‐2) via daily oral gavage for six consecutive days and subsequently euthanized on day 11 for further analysis (Figure 6a). Blood samples and major organs were harvested for further investigation, while body weight was monitored throughout the experimental period. No significant differences in body weight were observed between the health group and the sIHSELM‐treated group (Figure 6b). Furthermore, hematological analysis revealed that key blood cell parameters, including white blood cells, red blood cells, hematocrit, mean corpuscular volume, mean corpuscular hemoglobin, hemoglobin, and platelets, remained within normal physiological ranges in sIHSELM‐treated mice, with no statistically significant deviations compared to the health group (Figure 6c–i). Similarly, biochemical assessments of liver and kidney function demonstrated that serum levels of alkaline phosphatase (ALP), alanine aminotransferase (ALT), aspartate aminotransferase (AST), and blood urea nitrogen (BUN) were also within the normal limits, indicating preserved hepatic and renal functionality (Figure 6j–m). Finally, the hematoxylin and eosin (H&E) staining was conducted on major organs, including the heart, liver, spleen, lung, and kidney. All organs harvested from sIHSELM‐treated mice exhibited normal tissue architecture, with no discernible pathological alterations or inflammatory infiltrates compared to the health group (Figure S28). Additionally, the histological features of the gastrointestinal tract, including stomach, duodenum, jejunum, ileum, and colon, were investigated by both H&E and Alcian blue‐periodic acid Schiff (AB‐PAS) staining. These analyses confirmed that mucosal epithelial morphogenesis and overall tissue morphology remained unchanged in sIHSELM‐treated mice relative to healthy controls, with no evidence of cellular damage, inflammation, or goblet cell depletion (Figure 6n and Figure S29). These findings demonstrated the excellent biocompatibility and biosafety of sIHSELM, supporting its potential for clinical translation as a therapeutic agent for IBD and other diseases.

### 2.6 Bioremediation of sIHSELM

Microbial remediation exhibits distinct advantages over conventional remediation methods, primarily attributed to its cost‐effectiveness and high efficiency. Here, we chose organophosphate hydrolase (OPH) as a functional enzyme to achieve the bioremediation of sIHSELM. OPH demonstrates the ability to transform the hypertoxic paraoxon (PAR) into the irritant *para*‐nitrophenol (PNP), thereby mitigating the environmental and human health risks associated with PAR. Owing to these properties, OPH has been extensively employed as a biodegradation enzyme, either displayed or grafted onto ELMs, for bioremediation purposes [12, 17].

**FIGURE 7:**
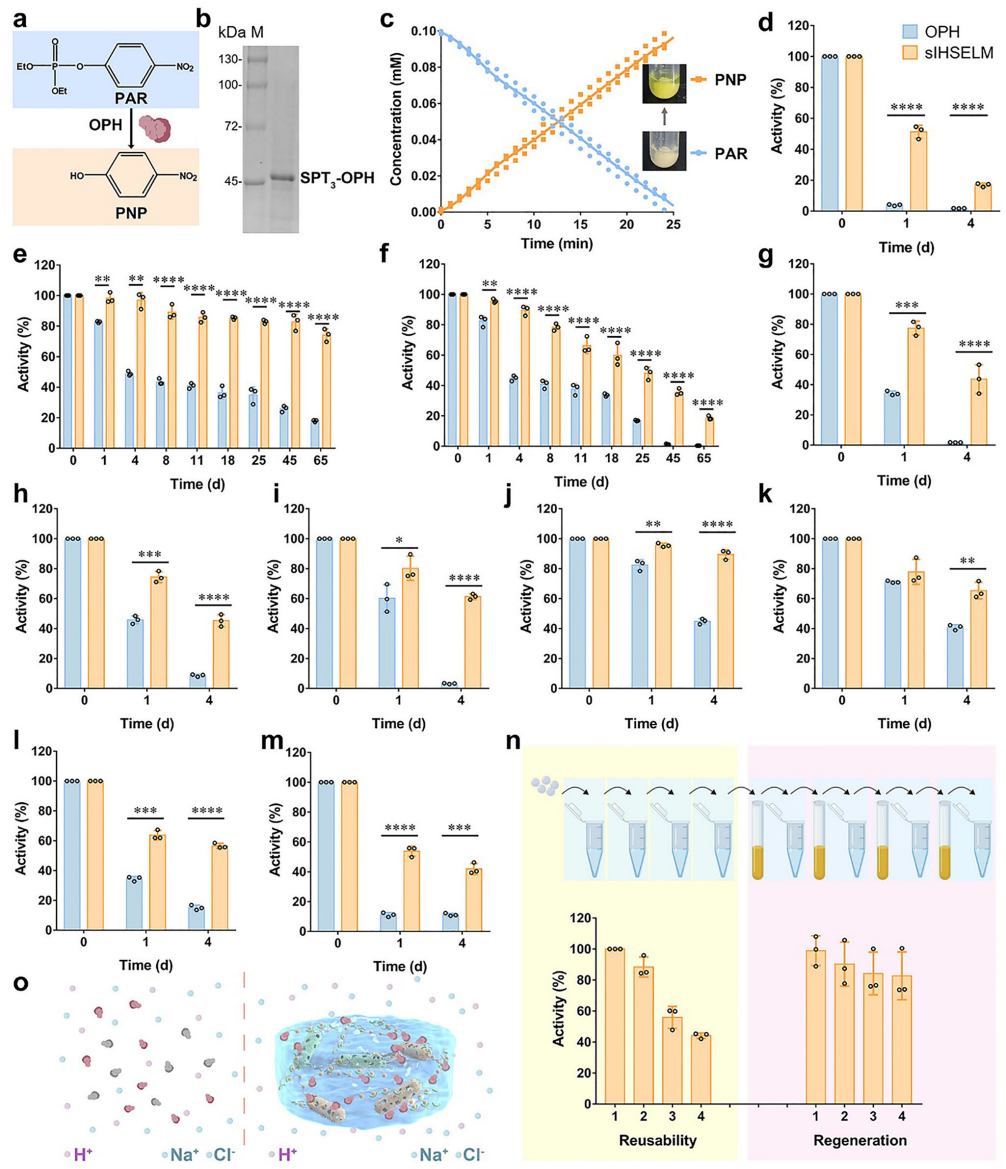
Bioremediation of sIHSELM. (a) OPH can convert the hypertoxic PAR into the irritant PNP. (b) The successful expression of SPT_3_‐OPH was verified via SDS‐PAGE. c Kinetics study of converting PAR to PNP utilizing sIHSELM. (d‐m) The relative enzyme activity of OPH, including free OPH and those encapsulated within sIHSELM, incubated at different temperatures, such as 42°C (d), 4°C (e) and 25°C (f), under different pH conditions, such as pH 4 (g), pH 5 (h), pH 6 (i) and pH 7 (j), and under different saline conditions, such as 20 g L^−1^ (k), 30 g L^−1^ (l) and 50 g L^−1^ (m) of NaCl. (n) The consecutive catalysis of PAR to PNP utilizing sIHSELM and the regeneration of catalytic activity. (o) Schematic representation of sIHSELM resistance to harsh environments. The patterns in (n) and (o) were created with BioGDP.com [52]. Data are presented as mean values ± SD (*n* = 3 biologically independent samples for (c–n)). ^*^ *p* < 0.05, ^**^ *p* < 0.01, ^***^ *p* < 0.001, ^****^ *p* < 0.0001.

As previously discussed, sIHSELM exhibited robust stability under varying pH conditions and elevated temperatures. Based on these characteristics, we replaced mCherry with OPH and fabricated the sIHSELM specifically for PAR biodegradation (Figure 7a). The successful expression of SPT_3_‐OPH was confirmed through SDS‐PAGE analysis (Figure 7b). Due to the high encapsulation capacity of bacteria within sIHSELM, the system demonstrated rapid conversion of PAR to PNP. 0.1 mM PAR was completely degraded into PNP within 25 min (Figure 7c), a rate surpassing that reported in the previous literature [17]. To assess the impact of encapsulation on OPH activity, we compared the activity of free OPH with that encapsulated within sIHSELM (with a comparable amount of OPH in both groups). The results indicated that the encapsulation of OPH had no significant effect on its activity (Figure S30). Furthermore, the findings from enzyme kinetic analysis corroborated this observation. As depicted in Figure S31, the *k*_cat_ values for both purified OPH and OPH encapsulated within sIHSELM were nearly identical, indicating that the encapsulation process had no significant influence on the intrinsic catalytic activity of OPH. However, a notable difference was observed in the Michaelis constant (*K*_m_) values. The *K*_m_ for sIHSELM‐encapsulated OPH increased to 0.69 ± 0.10 mm, in contrast to the *K*_m_ of 0.32 ± 0.04 mm for free OPH. This discrepancy is presumably attributable to the reduced accessibility of the PAR to the active sites of OPH within the sIHSELM matrix following encapsulation [62]. Despite the observed decrease in catalytic efficiency, the long‐term activity retention of OPH represents another critical issue that needs consideration for practical applications.

The activity of OPH is influenced by several factors, including pH and temperature [63, 64]. Notably, temperature has been regarded as one of the most formidable challenges confronting biological wastewater treatment techniques [65]. Therefore, we evaluated the enzymatic activity of OPH encapsulated within sIHSELM across a range of temperatures. To establish a baseline for comparison, free OPH was employed as a control, with its activity assessed under identical conditions to those applied to sIHSELM. When stored at 4°C, OPH encapsulated in sIHSELM exhibited remarkable long‐term stability, retaining approximately 80% of its initial activity over a period of 65 days (Figure 7e). However, the activity of free OPH declined precipitously from the fourth day, with only 18% of its original activity remaining after 65 days. Upon storage at 25°C, the activity of OPH within sIHSELM gradually diminished, yet it remained significantly higher than that of free OPH at equivalent time points. Specifically, around 50% of the initial activity was still detectable in the sIHSELM group within 25 days, whereas only 16% of activity persisted in the free OPH group (Figure 7f). Furthermore, when subjected to heating at 42°C for 4 days, the enzymatic activity in the sIHSELM group was markedly superior to that observed in the free OPH group (reduced to around 1.8%) (Figure 7d). This outstanding thermal stability of sIHSELM effectively shielded OPH from the detrimental effects of temperature fluctuations, thereby preserving its enzymatic function. These findings underscore the potential of sIHSELM as a robust platform for enhancing the stability and activity of OPH under varying thermal conditions.

The enzymatic activity of OPH exhibits an optimal performance at approximately pH 8 [66]. Notably, OPH retains sufficiently high activity even under elevated pH conditions (pH 9–10) [12, 17, 66]. However, under acidic to neutral conditions (pH 4–7), the activity of free OPH underwent a dramatic decline from the first day (Figure 7g–j), with a further decrease observed as acidity intensified. The activity of free OPH was almost completely lost within 4 days, plummeting to less than 2%. In contrast, when encapsulated within sIHSELM, OPH maintained approximately 43% of its activity even after incubation in acidic solutions (pH 4) (Figure 7g). The tolerance of OPH to high salinity also impacts its remediation efficiency, particularly in environments such as industrial wastewater, saline soils, and marine water. As expected, the activity of free OPH reduced drastically to less than 13% under high‐salinity conditions (30–50 g L^−1^ of NaCl) within 4 days. In contrast, OPH encapsulated in sIHSELM maintained 42%‐56% of its activity under identical conditions (Figure 7k–m). Furthermore, the engineered sIHSELM demonstrated remarkable reusability, retaining around 45% of its initial activity after four consecutive rounds of catalyzing PAR (Figure 7n). The sIHSELM could be regenerated by immersion in LB medium, after which OPH activity was restored to nearly 100%, with only slight reductions observed after four regeneration cycles (Figure 7n). This phenomenon could be attributed to the fact that the encapsulated bacteria underwent regeneration and subsequently synthesized new OPH enzymes, thereby restoring the catalytic activity of sIHSELM. These findings collectively underscored the capacity of sIHSELM to serve as a protective harbor for OPH, shielding it from harsh environmental conditions, including thermal, pH, and saline stresses (Figure 7o). The sustained enzymatic activity of OPH within sIHSELM over extended periods and the regeneration of sIHSELM render it a promising candidate for the treatment of complexly polluted water, offering a robust and reusable platform.

## 3 Discussion

We have successfully developed a universal sIHSELM fabrication strategy, which not only enables precise expression of customized functional proteins but also demonstrates broad cross‐disciplinary applicability, thereby contributing additional perspectives to biomaterials science.

First, we developed an sIHSELM platform that integrated the thermoresponsive properties of HBC hydrogels with a synthetic biology‐mediated release‐assembly mechanism. HBC, a thermosensitive material exhibiting excellent water solubility and rapid sol‐gel phase transition capabilities, has not been explored for ELM fabrication. In our study, HBC was utilized for bacterial encapsulation, providing a supportive and protective environment for the bacteria. Concomitantly, Spy proteins released from the encapsulated bacteria underwent self‐polymerization to form a semi‐IPN structure within the HBC matrix to enhance the stability and mechanical strength of sIHSELM. In conventional semi‐IPN architectures, the chemically crosslinked polymer component provides a rigid structural framework and contributes to high modulus, while the physically entangled linear polymer component enables effective energy dissipation through reversible chain segment mobility. This synergistic combination of rigid and ductile phases allows for tailored mechanical reinforcement. In this study, the semi‐IPN structures, composed of HBC physical networks and Spy protein self‐assembled networks, overcame the inherent limitations of both pure HBC hydrogels and naturally derived protein gels [67, 68]. Notably, sIHSELM exhibited dynamic living characteristics that distinguished it from conventional semi‐IPNs. The continuous biosynthesis of Spy proteins by viable bacteria enabled sustained structural reinforcement and uninterrupted functionality. Additionally, by rigorously modulating the encapsulated bacterial load, an optimal balance was established between the structural reinforcement conferred by semi‐IPNs formation and the structural disruption induced by bacteria encapsulation, which was crucial for maintaining the integrated morphology and enhancing the mechanical robustness of sIHSELM.

Second, sIHSELM demonstrates exceptional environmental stability. sIHSELM could maintain structural and functional integrity across a broad pH range (pH 2–12), elevated temperatures (up to 42°C), and saline conditions (up to 50 g L^−1^ of NaCl). Furthermore, sIHSELM could maintain its intact shape under different pH conditions at room temperature for two years (Figure S32), indicating its exceptional long‐term storage stability. In contrast, current ELM materials exhibit critical limitations. For instance, Pluronic F‐127, PVA, and PEO hydrogels show rapid dissolution in aqueous environments [16]. Ca^2+^‐crosslinked alginate hydrogels undergo accelerated degradation when exposed to liquid environments [69]. Collagen/gelatin‐based hydrogels suffer from inferior mechanical properties and thermal instability [70]. Tripolyphosphate‐crosslinked chitosan microcapsules exhibit pH‐dependent aggregation and ionic strength sensitivity [71]. Compared to these ELMs, sIHSELM demonstrates improved operational stability. Notably, this robust stability creates a protected microenvironment for encapsulated bacteria, shielding them from external disturbances while maintaining the functionality of the proteins. For example, IL‐2‐expressing sIHSELM demonstrated a pronounced therapeutic efficacy in alleviating acute colitis, attributable to the protection of ECN in the gastrointestinal tract. ECN could be delivered to the intestine and release IL‐2, facilitating precise modulation of pro‐/anti‐inflammatory cytokine release, innate immune responses, and gut microbiota composition. In the other case, OPH‐expressing sIHSELM exhibited prolonged PAR biodegradation capabilities under harsh conditions due to the OPH activity maintenance within sIHSELM. In contrast, free OPH exhibited significant activity loss under acid (pH 4–6), thermal (25–42°C), and saline (30–50 g L^−1^ of NaCl) stress conditions. The protective efficacy of OPH within the sIHSELM not only matches but, under specific conditions, surpasses that achieved through immobilization strategies employing porous fabric materials, nanoscaffolds, metal nanoparticles, or surface display on bacteria [62, 72, 73, 74, 75, 76].

Third, sIHSELM exhibits safety, moldability, and ease of fabrication. Owing to the excellent biocompatibility and water solubility of HBC, the dissolution and gelation of HBC hydrogels do not necessitate the use of acidic, alkaline, or chemical crosslinking agents, thereby ensuring the safety of sIHSELM. The fabrication process of sIHSELM is remarkably straightforward, requiring only the resuspension of bacteria in HBC solutions followed by thermal gelation, without the need for complex protein purification steps. When compared to photo‐crosslinking hydrogels such as PEGDA and GelMA, the advantages of sIHSELM are fully demonstrated. The ultraviolet irradiation required for crosslinking may exert detrimental effects on the encapsulated bacteria, limiting their innate metabolism and protein expression [77]. The fabrication process of sIHSELM is environmentally sustainable, has potential for scalable production, and is mild enough to avoid adverse effects on the encapsulated bacteria. Owing to the stable thermo‐responsive gelation properties of HBC, the fabricated sIHSELM showed no significant variation across different production batches (Figure S33), underscoring its batch‐to‐batch consistency. More importantly, benefiting from the excellent thermosensitive and 3D‐printable properties of HBC [78], sIHSELM can be fabricated into various desired morphologies, including both microscale and macroscale forms.

Fourth, sIHSELM demonstrates high customizability and modularity, allowing for the integration of diverse gene circuits, bacterial strains, functional proteins, and even shape designs to meet specific application requirements. For example, we employed two *E. coli* strains (BL21(DE3) and ECN) to express two distinct functional proteins (OPH and IL‐2) under two types of induced lysis conditions (temperature and arabinose) by utilizing synthetic biology for two separate applications (bioremediation and biomedical therapy). More importantly, we attempted to enhance the modularization of sIHSELM by encapsulating three bacterial strains. Given the low expression level of IL‐2 in prokaryotes and the requirement for intact and non‐fused IL‐2 to exert its biological function, bacteria expressing IL‐2 were co‐encapsulated with those expressing two Spy proteins within sIHSELM. By regulating the loading of the three bacteria, both the intact conformation and function were preserved. This result substantially expands the applicability of sIHSELM in scenarios involving low expression of active proteins.

In summary, by reprogramming the functional protein payloads, the sIHSELM platform can be adapted for a wide range of other applications, including biosensing, bioenergy production, and biocatalysis. sIHSELM can undergo surface modification using polyacrylamide or chitosan to prevent bacteria from escaping and thereby enhance its biosafety [10, 19]. Nevertheless, plasmid instability remains a critical factor that may compromise the long‐term functionality of sIHSELM (Figure S34). The functional gene expression cassette can be further optimized through genome integration, thereby enhancing genetic stability. Additionally, multifunctional genes with cascade functions can be designed to co‐express in the sIHSELM platform to expand its applications in complex scenarios. Therefore, further studies are ongoing to address these issues.

## 4 Conclusion

By innovatively constructing a bacteria‐hydrogel synergistic system, we encapsulated engineered bacteria expressing the Spy proteins (SpyCatcher and SpyTag tri‐block protein) within a highly biocompatible HBC matrix. This approach facilitated intelligent controlled release and self‐assembly of Spy proteins, leading to the establishment of an environmentally stable, biocompatible, and easily prepared sIHSELM platform. Guided by synthetic biology design principles, we incorporated IL‐2 and OPH into this platform, respectively, to construct functionally specialized ELMs. The results revealed that the IL‐2‐expressing sIHSELM exhibited remarkable therapeutic efficacy in a mouse colitis model. In the other case, the OPH‐expressing sIHSELM maintained exceptional enzymatic activity stability under harsh conditions for extended periods, including high temperature, extreme pH, and high salinity, overcoming the application limitations of traditional biocatalysts. These advancements establish a comprehensive framework for fabricating a universal ELM platform with customized functionalities. Moreover, they offer new perspectives for developing functional ELMs in bioremediation and biotherapeutics applications.

## Funding

This work was partially supported by grants from the Shandong Provincial Natural Science Foundation (ZR2024ME226), the Natural Science Foundation of Qingdao (23‐2‐1‐170‐zyyd‐jch), Young Scholars Program of Shandong University (Z.X.B.), the Taishan Scholars Program (tstp20231208), and the SKLMT Frontiers and Challenges Project (SKLMTFCP‐2023‐03).

## Conflicts of Interest

The authors declare no conflict of interest.

## Supporting information




**Supporting File**: advs74803‐sup‐0001‐SuppMat.pdf

## Data Availability

The data that support the findings of this study are available from the corresponding author upon reasonable request.
