# Supplementary material for: Programmable Semi‐Interpenetrating Living Materials With Robust Stability for Versatile Bioremediation and Biotherapeutics
Source: Adv Sci (Weinh). 2026 Mar 13;13(29):e24320. doi: 10.1002/advs.202524320 (PMC13205668; doi:10.1002/advs.202524320)
Supplement: Supplementary file 1 — Supporting File: advs74803‐sup‐0001‐SuppMat.pdf [file ADVS-13-e24320-s001.pdf]

Supporting Information

Programmable semi-interpenetrating living materials with robust stability for versatile bioremediation and biotherapeutics

Zixian Bao[1], Shengfeng Yang[2], Dandan Hu[1], Jiezheng Liu[1,3], Bo Jiang[1], Xinyue Sui[1], Qingsheng Qi[1], Lai Li [2,], and Guang Zhao[1,]**

[1] State Key Laboratory of Microbial Technology and Institute of Microbial Technology, Shandong University, Qingdao 266237, China

[2] Qingdao Central Hospital, University of Health and Rehabilitation Sciences (Qingdao Central Hospital), Qingdao 266042, China

[3] CAS Key Laboratory of Biobased Materials, Qingdao Institute of Bioenergy and Bioprocess Technology, Chinese Academy of Sciences, Qingdao, 266101, China

* Correspondence: llzsm@163.com (L.L.); zhaoguang@sdu.edu.cn (G.Z.)

1. Methods

1.1 Materials

Chitosan (viscosity 72 cps) with a deacetylation degree of 90% was purchased from Laizhou Haili Biological Product Co., Ltd. (Laizhou, China). Trypsin and pepsin were purchased from Solarbio (Beijing, China). All other reagents were of analytical grade.

1.2 Cell strains, plasmids and animals

The *E. coli* strain BL21(DE3) was purchased from Beijing Tsingke Biological Technology Co., Ltd. (Beijing, China), and *E. coli* Nissle 1917 (ECN) was purchased from HonorGene (Changsha, China). BL21(DE3) and ECN were cultured in Luria-Bertani (LB) medium with shaking at 37 °C and 200 rpm. φX174E, SpyCatcher, SpyTag, OPH, and IL-2 genes with codon optimization were synthesized from Beijing Tsingke Biological Technology Co., Ltd. (Beijing, China). φX174E gene was cloned into pBAD and pBV220 vectors using a restriction enzyme-mediated cloning approach to construct pBAD-φX174E and pBV220-φX174E plasmids, respectively. pACYCDuet-SPC_3 (SpyCatcher-ELP-SpyCatcher-ELP-SpyCatcher) and pACYCDuet-SPT_3-mCherry (SpyTag-ELP-SpyTag-ELP-SpyTag-mCherry) plasmids were constructed by inserting the coding gene into pACYCDuet-1 vector, in which ELP was fused with either multiple SpyCatcher or SpyTag sequences. To achieve the expression of Spy proteins in ECN, the promoter of pACYCDuet-1 vector was replaced with FNR promoter, and pACYCDuet-P_{FNR}-SPC_3 and pACYCDuet-P_{FNR}-SPT_3-mCherry plasmids were constructed. IL-2 gene with N-terminal LamB peptide and C-terminal InfB tag was cloned into pACYCDuet-1 vector with FNR promoter to construct pACYCDuet-P_{FNR}-LamB-IL-2-InfB plasmid. All constructed plasmids were confirmed by DNA sequencing (Tsingke, China).

The pACYCDuet-SPC_3 and pACYCDuet-SPT_3-mCherry plasmids were transformed into BL21(DE3) to obtain BL21-SPC_3 and BL21-SPT_3-mCherry strains, respectively. Subsequently, pBV220-φX174E plasmid was transformed into BL21-SPC_3 and BL21-SPT_3-mCherry strains, respectively. pACYCDuet-P_{FNR}-SPC_3, pACYCDuet-P_{FNR}-SPT_3-mCherry and pACYCDuet-P_{FNR}-IL-2 plasmids were transformed into ECN to obtain ECN-SPC_3, ECN-SPT_3-mCherry and ECN-IL-2, respectively. pBAD-φX174E was then transformed into ECN-SPC_3 and ECN-SPT_3-mCherry, respectively. The resulting BL21-φX174E/SPC_3, BL21-φX174E/SPT_3-mCherry,

ECN-φX174E/SPC_3 and ECN-φX174E/SPT_3-mCherry strains were utilized for the fabrication of ELMs.

Female C57BL/6 mice (6-8 weeks) were housed and cared under a photoperiod schedule of 12 h light-dark cycle, and the temperature was maintained within the range of 20 to 26 °C, with a humidity of 40-70%. Moreover, they were provided with free access to food (GB 14924.3-2010 feed formula) and water. All animal experiments were performed in compliance with the relevant laws and approved by the Institutional Animal Care and Use Committee of Shandong University (No. SYDWLL-2024-133).

1.3 Protein expression and purification

BL21-SPC_3 and BL21-SPT_3-mCherry strains were cultured with LB medium in an oscillating incubator at 37 °C until the optical density (OD) at 600 nm reached 0.6-0.8. Afterward, IPTG (0.1 mM) was added to induce the expression of the SPC_3 and SPT_3-mCherry protein. Following induction at 30 °C for 6 h, the cells were harvested via centrifugation, and disrupted using a high-pressure cell disruptor (Constant Systems LTD, UK). After centrifugation, the supernatant was harvested. Proteins were purified with Ni-NTA and detected using SDS-PAGE with Coomassie Blue stain.

1.4 *In vitro* and *in vivo* reaction between SPC_3 and SPT_3-mCherry proteins

For the *in vitro* reaction, purified SPC_3 and SPT_3-mCherry proteins were mixed at mole ratios of 1:1 and 1:2 (SPC_3:SPT_3-mCherry), which were incubated at 37 °C with a shaking speed of 100 rpm for 24 h. At a predetermined time, samples were withdrawn and subjected to boiling at 100 °C for 10 min to terminate the reaction.

Regarding the *in vivo* reaction, the BL21-φX174E/SPC_3 and BL21-φX174E/SPT_3-mCherry strains were cultured with LB medium in an oscillating incubator at 37 °C until the $OD_{600\ nm}$ reached 0.6-0.8, respectively. Protein expression was induced by adding IPTG (0.1 mM). The BL21-φX174E/SPC_3 and BL21-φX174E/SPT_3-mCherry cells were cultured at 30 °C for 3 h, respectively, and subsequently mixed together at a CFU ratio of 1:1.5. After cultured at 42 °C for 12 h, the supernatant was harvested via centrifugation and the proteins were purified using Ni-NTA.

The *in vitro* and *in vivo* reaction between SPC_3 and SPT_3-mCherry proteins were detected

using SDS-PAGE with Coomassie Blue stain.

1.5 The bacterial lysis and responsive release of Spy proteins

To assess the comparative lysis efficiency under temperature and arabinose induction conditions, BL21-pBV220-φX174E/SPT_3-mCherry and BL21-pBAD-φX174E/SPT_3-mCherry strains were cultured with LB medium in an oscillating incubator at 37 °C. Bacteria that carried the same vectors without φX174E gene were employed as the control groups. When the $OD_{600\ nm}$ reached 0.6-0.8, IPTG (0.1 mM) was administered to all groups. Additionally, arabinose (0.2 wt%) was added to the BL21-pBAD-φX174E/SPT_3-mCherry group. Subsequently, bacteria carrying pBV220 vectors were cultured at 42 °C for 21 h, whereas those harboring pBAD vectors were cultured at 30 °C for 21 h. At predetermined time, the $OD_{600\ nm}$ was measured, and bacterial samples were collected, diluted at various gradients, and enumerated using the plate count method.

The release of Spy proteins was induced by either temperature (42 °C) or arabinose. BL21-φX174E/SPT_3-mCherry or ECN-φX174E/SPT_3-mCherry strain was cultured with LB medium in an oscillating incubator at 37 °C. Regarding the BL21-φX174E/SPT_3-mCherry strain, when the $OD_{600\ nm}$ reached 0.6-0.8, IPTG (0.1 mM) was added and the culture was continued at 30 °C for 3 h. Subsequently, the culture was transferred to 42 °C and continued for 24 h. Regarding the ECN-φX174E/SPT_3-mCherry strain, after cultured at 37 °C for 6 h, arabinose (0.2 wt%) was added and the culture was continued for 24 h. At predetermined time, samples of the culture were taken and centrifuged at 10000 *g* for 5 min. The fluorescence intensity of mCherry in the culture and supernatant was determined using a multimode microplate reader (Spark, Tecan) with an excitation wavelength of 588 nm and an emission wavelength of 645 nm.

1.6 Preparation of HBC

HBC was prepared as described previously [1]. Briefly, chitosan was subjected to mixing with NaOH, followed by stirring for 24 h. Subsequently, the excess NaOH solution was removed. Thereafter, the chitosan was dispersed in an isopropanol-water mixture and subsequently reacted with 1,2-butene oxide at a temperature of 55 °C for 24 h. Finally, the resulting product was neutralized, dialyzed against distilled water, and then lyophilized. The degree of substitution was measured with elemental analysis method [2]. The percentages of C and N

elements in chitosan and HBC were measured, and the degree of hydroxybutyl substitution per residue for HBC was calculated as 1.57 with a known molecular formula [3]. The molecular weight of HBC was 322 kDa, which was measured with gel permeation chromatography. The concentration of HBC used in all experiments was 5 wt%.

1.7 Fabrication of sIHSELM

Lyophilized HBC was sterilized by UV irradiation, and then dissolved in sterilized PBS solution (0.01 M, pH 7.4) at 4 °C overnight to obtain homogeneous HBC solutions (5 wt%). BL21-φX174E/SPC3 and BL21-φX174E/SPT3-mCherry strains were cultivated in LB medium within an oscillating incubator at 37 °C. Once the $OD_{600\ nm}$ reached 0.6-0.8, IPTG (0.1 mM) was added, and the cultivation was continued at 30 °C for 6 h. These strains were mixed at CFU ratios of 10:15, 20:30 and 30:45 (BL21-φX174E/SPC3: BL21-φX174E/SPT3-mCherry), and then collected via centrifugation at 5000 rpm for 10 min. After washed with PBS for three times, the strains were resuspended with 0.5 mL of HBC solutions. Subsequently, this suspension was incubated at 42 °C for 6 h to prepare the sIHSELM, designated as $sIHSELM_{C10+T15}$, $sIHSELM_{C20+T30}$, $sIHSELM_{C30+T45}$, respectively. For samples containing only one type of Spy protein, BL21-φX174E/SPC3 or BL21-φX174E/SPT3-mCherry strains (with CFUs equivalent to those of the corresponding sIHSELM samples) were incorporated into HBC solutions to prepare HSELM, designated as $HSELM_{C25}$, $HSELM_{T25}$, $HSELM_{C50}$, $HSELM_{T50}$, $HSELM_{C75}$, and $HSELM_{T75}$, respectively.

1.8 Fabrication of sIHSELM for PAR degradation

BL21-φX174E/SPC3 and BL21-φX174E/SPT3-OPH strains were cultivated in LB medium within an oscillating incubator at 37 °C. Once the $OD_{600\ nm}$ reached 0.6-0.8, IPTG (0.1 mM) was added, and the cultivation was continued at 30 °C for 6 h. These strains were mixed at a CFU ratio of 20:30 (BL21-φX174E/SPC3: BL21-φX174E/SPT3-OPH), and then harvested via centrifugation at 5000 rpm for 10 min. After washed with PBS for three times, the strains were resuspended with 0.5 mL of HBC solutions. Subsequently, this suspension was slowly dripped into 150 mL of preheated olive oil under stirring to obtain microspheric sIHSELM. The microspheres were incubated at 37 °C for 10 min, then transferred to 42 °C and continued for 8 h to prepare the sIHSELM used for PAR degradation.

1.9 Fabrication of sIHSELM for IBD therapy

ECN-φX174E/SPC$_3$ and ECN-φX174E/SPT$_3$-mCherry strains were cultivated in LB medium within an oscillating incubator at 37 °C with a shaking speed of 180 rpm for 6 h. ECN-IL-2 strain was cultivated in LB medium within an oscillating incubator at 37 °C with a shaking speed of 180 rpm for 3 h. These strains were mixed at CFU ratios of 20:20:10 (ECN-φX174E/SPC$_3$: ECN-φX174E/SPT$_3$-mCherry: ECN-IL-2), and then collected by centrifugation at 5000 rpm for 10 min. After washed with PBS for three times, the strains were resuspended with 0.5 mL of HBC solutions. Subsequently, this suspension was slowly dripped into 150 mL of preheated olive oil under stirring, and then incubated at 37 °C for 10 min to obtain microspheric sIHSELM. The sIHSELM were collected by centrifugation, and incubated with LB medium containing 0.2% arabinose at 37 °C for 6 h to prepare the sIHSELM used for IBD therapy.

1.10 Western blot

Protein samples were separated by a 12% sodium dodecyl sulfate polyacrylamide gel and subsequently transferred onto a PVDF membrane (Millipore). The PVDF membrane was blocked for 2 h at room temperature using quick block Western reagent (Beyotime, China). Thereafter, the membrane was incubated overnight at 4 °C with the primary antibody against IL-2 (26156-1-AP, Proteintech, 1:1000). Following the incubation, the PVDF membrane was washed 5 times with TBST (Tris-buffered saline containing 0.1% Tween® 20), and then incubated with the HRP-conjugated anti-rabbit secondary antibody (A01827, GenScript Biotech) with a dilution ratio of 1:5000. Finally, the protein bands were detected using a Fusion FX6 Imaging System (Vilber, France).

1.11 Characterization

The cross-sectional microstructures of lyophilized sIHSELMs were captured with a field-emission SEM (Quanta 250 FEG, FEI, USA) at 5 kV. The thermal stability of sIHSELMs was assessed via TGA. The TGA measurements were performed using a thermogravimetric analyzer (TG 209F3, Netzsch, Germany) with a heating rate of 20 °C min^{-1}, within a temperature range of 30-800 °C, and under a nitrogen atmosphere flowing at a rate of 20 mL min^{-1}. The mechanical strength of sIHSELMs was evaluated through compression tests using an Instron 5543 universal

tensile machine (Instron Corp., Canton, USA). Cylindrical specimens were compressed at a constant stress rate of 20 mN min^{-1}. The rheological properties of HBC and sIHSELM hydrogels were evaluated using a rotational rheometer (Anton Paar-702e, Anton Paar Ltd, Austria) equipped with a parallel plate geometry (0.1 mm gap). The storage modulus (G′) and loss modulus (G″) of HBC hydrogels were determined across a temperature range from 4 °C to 40 °C at a constant heating rate of 1 °C min^{-1}. The gelation temperature and gelation time were defined as the temperature and time, respectively, at which G′ and G″ were equivalent in value. For sIHSELM hydrogels, the measurements of G′ and G″ were recorded under two different conditions: either within a frequency range of 0.1 Hz to 10 Hz while maintaining a constant strain of 1%, or within a strain range of 0.1% to 10% while keeping the frequency constant at 1 Hz. The distribution of SPT_3-mCherry protein within sIHSELM hydrogels was visualized using confocal laser scanning microscopy (TCS SP8, Leica, Germany). The live and dead bacteria within sIHSELM hydrogels (which expressed SPT_3-OPH instead of SPT_3-mCherry) were stained using LIVE/DEAD™ BacLight™ Bacterial Viability Kits (L7012, Invitrogen, Thermo Fisher Scientific, USA) and subsequently observed via fluorescence microscopy (CKX53, Olympus, Japan).

1.12 Chitosanase degradation on sIHSELM

The as-prepared HBC and sIHSELM hydrogels were subjected to incubation with 1 mL of chitosanase (1 U mL^{-1}, Shanghai Yuanye Biotechnology Co., LTD, China) in 50 mM sodium acetate buffer (pH 4.5) at 37 °C. The weights of hydrogels in both groups were measured at predetermined time. Following a 4-hour incubation period, the residual hydrogels were carefully removed, lyophilized, and subsequently examined using SEM (Quanta 250 FEG, FEI, USA).

1.13 The stability of sIHSELM

sIHSELMs were weighted and incubated with different pH solutions (pH 2, pH 7 and pH 12), simulated gastric fluid (SGF, pH 2.5 containing 10 mg mL^{-1} of pepsin in 0.85% NaCl solution) or simulated intestinal fluid (SIF, pH 6.8 containing 10 mg mL^{-1} of trypsin and 0.5 M KH_2PO_4) at 37 °C for 14 days. At the predetermined time, the specimens were taken out and rinsed three times with deionized water. The washed hydrogels were gently blotted dry until they reached a

constant weight and then weighed. The mass loss ratios were calculated as the ratio of the remaining weight to the initial weight of the hydrogels.

1.14 The protection of sIHSELM *in vitro*

To investigate the protective effect of sIHSELM on engineered strains, ECN or sIHSELM were incubated in SGF or SIF and incubated at 37 °C for 2 h. Subsequently, the bacterial samples were collected, washed, and diluted at various gradients. Following this, they were spread onto solid agar plates supplemented with chloramphenicol. After 24 h of incubation, the number of bacteria was counted.

ECN or sIHSELM were stored at -80 °C for 8 days. At the predetermined time, these ECN or sIHSELM were slowly thawed on ice, diluted at various gradients, and the number of bacteria was counted using the plate count method.

1.15 *In vitro* release of IL-2 from sIHSELM

sIHSELM microspheres expressing IL-2 (containing 1×10^9 CFU of ECN-IL-2) were incubated in 3 mL of SIF within an oscillating incubator at 37 °C. At predetermined time, samples (100 μL) were withdrawn and subsequently centrifuged at 12000 rpm for 2 min. The resulting supernatant was then utilized for the detection of IL-2. The concentration of expressed and released IL-2 was quantified using a commercial ELISA kit (Beyotime PI575, China) in strict accordance with the manufacturer's instructions.

1.16 Therapeutic effect against IBD model

1.16.1 Animal treatment

Female C57BL/6 mice were randomly allocated into four groups, each consisting of five mice. Throughout the first six days, the mice were given drinking water containing 3% DSS to induce colitis, except for the healthy group. After that, they were gradually shifted back to their normal drinking water. On designated days, the mice were administered PBS, ECN-IL-2 (1×10^9 CFU), or sIHSELM (with comparable 1×10^9 CFU of ECN-IL-2) via oral gavage. Throughout the entire period, the body weights were meticulously monitored every day. On day 11, all groups of mice were euthanized, and the distal colon tissues were collected for further evaluations.

1.16.2 DAI assessment

Following the completion of the IBD treatment, the viscosity and presence of fecal blood were meticulously observed in mice. The parameters employed for the assessment of DAI were derived from a prior publication [4], and were assigned appropriate scores. As depicted in Table S2, the parameters representing the DAI were recorded, and the DAI is calculated as the sum of the scores of various parameters.

1.16.3 Histological analysis

The harvested distal colons of mice in each group were fixed in a 4% paraformaldehyde (PFA) solution for 24 h. Subsequently, they underwent dehydration and were embedded in paraffin for subsequent sectioning. Following this, H&E staining was conducted. The stained sections were then observed using an optical microscope (Nikon Eclipse Ci-L, Japan). The histological damage of the colon was evaluated in a blinded manner according to a previously established set of recommended criteria [5]. The corresponding scores for different parameters are presented in Table S3. Colonic damage score is calculated as the sum of the scores of various parameters.

1.16.4 Immunofluorescence staining

The harvested distal colons of mice in each group were fixed in a 4% PFA solution for 24 h. The fixed colon tissue was subsequently sectioned and incubated with primary antibodies, including anti-ZO-1 (Solarbio K001718P, 1:500) and anti-Occludin (Proteintech 27260-1-AP, 1:500), at 4 °C for 12 h. After washing with PBS for three times, the colon sections were then stained with AF488 anti-rabbit secondary antibody (Beyotime P0176, 1:1000) or AF555 anti-rabbit secondary antibody (Beyotime P0179, 1:1000) at room temperature for 2 h. After an additional three washes with PBS, the sections were incubated in DAPI at room temperature for 1 h in the dark. Ultimately, the colon sections were visualized using a confocal laser scanning microscope (Zeiss LSM880, Germany), and fluorescence signals were quantified with ImageJ software.

1.16.5 Flow cytometry analysis

The harvested colon tissues were homogenized using a tissue grinder, resulting in the formation

of a cellular suspension. The resultant suspension was washed with cold PBS for three times, and the surface staining was achieved by using a combination of mouse monoclonal antibodies conjugated with fluorochromes, including PECY5-CD45 (BioLegend 103110), FITC-CD11B (BioLegend 101206), PE-LY6G (BD 551461), FITC-CD3 (BioLegend, 100204), and PECY7-CD4 (BioLegend, 100528). The staining was carried out at room temperature for 30 min.

For intracellular staining, the cells were fixed in 4% PFA at room temperature for 30 min. After washing for twice times, the cells were incubated with PE-Foxp3 (BioLegend 126404) and APC-IL-17a antibodies (BioLegend, Cat. No. 506916) at room temperature for 20 min. Analysis of the stained cells was performed using a flow cytometer (DAKEWE EXFLOW206, China) in conjunction with FlowJo software.

1.16.6 ELISA analysis

To assess the concentrations of inflammatory cytokines, the harvested colon tissues were homogenized using a tissue grinder. Subsequently, the obtained samples were centrifuged at 12000 rpm for 20 min at 4 °C to isolate the supernatant. Following the quantification and normalization of proteins using the BCA kit, the concentrations of inflammatory cytokines, including TNF-α, IL-6, IL-1β, TGF-β1 and IL-10 were measured using ELISA kits (Invitrogen, Thermo Fisher Scientific, USA) in strict accordance with the manufacturer's instructions.

1.16.7 Gut microbiota 16S sequencing assay

After different treatments, the feces of mice were harvested, frozen in liquid nitrogen, and sent to BGI Genomics Co., Ltd. for 16S sequencing assay. The total genomic DNA of samples was extracted, and the DNA concentration was measured using the Qubit® dsDNA HS Assay Kit. The MetaVX Library Preparation Kit was utilized to construct the sequencing library. A panel of primers (338F: ACTCCTACGGGAGGCAGCAG, and 806R: GGACTACHVGGGTWTCTAAT), specific to the V3 and V4 hypervariable regions of the microbiota 16S rDNA, were adopted for the construction. Next-generation sequencing was carried out on an Illumina Miseq/Novaseq Platform (Illumina, San Diego, USA) at GENEWIZ.

16S rDNA gene sequencing analysis was conducted using the QIIME 2 data analysis package. In brief, the forward and reverse reads were joined and assigned to samples based on the barcode. Subsequently, the barcode and primer sequences were further removed. The

resulting product was filtered to delete sequences containing ambiguous bases, those with a length exceeding 200 bp, or those with a mean quality score lower than 20. Chimeric sequences were identified using the UCHIME algorithm with reference to the RDP Gold database and then discarded to obtain the effective sequences for final analysis. The clustering program VSEARCH (1.9.6) was employed to cluster the sequences into operational taxonomic units (OTUs) at a 97% sequence identity level. The 16S rDNA reference database used was Silva 132. In accordance with the findings of the OTU analysis, subsequent computations were performed to ascertain the Shannon diversity index, Chao1 alpha diversity index, and the abundance of community species.

1.17 *In vivo* toxicity analysis

Female C57BL/6 mice were randomly allocated into two groups, each consisting of five mice. On designated days, the mice were administered PBS, or sIHSELM (1×10^9 CFU of ECN-IL-2) via oral gavage. On day 11, all groups of mice were euthanized. Blood samples were obtained for hematological and biochemical assessments. Major organs, including heart, liver, spleen, lung and kidney, were harvested for histopathological evaluation via H&E staining. Additionally, gut samples, including colon, ileum, jejunum, duodenum and stomach, were also collected for histological analysis via H&E and AB-PAS stains. The stained sections were observed using an optical microscope (Nikon Eclipse Ci-L, Japan).

1.18 Biodegradation by sIHSELM

OPH-expressed sIHSELM was prepared and resuspended in 0.1 M CHES buffer (pH 7.4) with 50 mM $CoCl_2$, which was incubated at 30 °C for 1 h. Subsequently, sIHSELM was immersed in 200 μL of CHES buffer (0.1 M, pH 10) containing 20 μL of PAR (3 mM in 25% methanol). The biodegradation was maintained at 37 °C for 30 min. The calibration curve was generated using standard PNP solutions. The concentration of PNP was determined by measuring the absorbance at 405 nm every minute using a multimode microplate reader (Spark, Tecan). The consumption of PAR was calculated based on the generation of PNP.

To verify that the OPH activity was not affected during the preparation of sIHSELM, sIHSELM and purified OPH (with a comparable amount of OPH in both groups) were employed to degrade PAR in the same conditions as described aforementioned. The activity of

each group was normalized against the activity of purified OPH.

Regarding kinetic parameters, purified OPH and sIHSELM expressing OPH containing the equivalent amount of proteins (0.13 μg) were utilized. The final concentration of PAR in the reaction solution varied from 0.05 mM to 6 mM. All reaction conditions were meticulously maintained according to the aforementioned OPH activity assay method. Subsequently, the collected data were analyzed by fitting them into the Michaelis-Menten equation, $v = V_{max} \times [S] / (K_m + [S])$, to derive the kinetic constants. In this equation, v represents the initial reaction velocity, V_{max} denotes the maximum reaction velocity, $[S]$ stands for the concentration of PAR, and K_m signifies the Michaelis constant. Furthermore, the catalytic efficiency of the enzyme was quantified by calculating the k_{cat}/K_m ratio, wherein k_{cat} was derived using the formula $k_{cat} = V_{max}/[E]_T$, with $[E]_T$ representing the total concentration of the OPH enzyme.

For reuse experiments, sIHSELM was immersed in PAR-CHES solutions (as described aforementioned) for 2 h, and the process was repeated 4 times.

For regeneration experiments, sIHSELM was immersed in LB medium (containing 0.1 mM of IPTG) at 37 °C for 12 h, and then incubated in PAR-CHES solutions (as described aforementioned) for 2 h. The process was repeated 4 times.

1.19 The protection of OPH activity in sIHSELM

OPH-expressed sIHSELM was prepared as described in the previous section. sIHSELM and purified OPH were incubated at 4 °C and 25 °C for 65 days, and at 42 °C for 4 days. At predetermined time, the sIHSELM and purified OPH (with comparable amount of OPH in the two groups) were taken out and the biodegradation of PAR was assessed in the same conditions as described aforementioned. The activity of each group was calculated by taking the time derivative of PNP concentration. The activity determined at each time point was normalized against the original activity before incubation (0 day).

To assess the protection of OPH activity in an acid environment, sIHSELM and purified OPH were incubated under different pH solutions (pH 4, pH 5, pH 6, pH 7) at 25 °C for 4 days. The OPH activity was evaluated and calculated as described aforementioned.

To assess the protection of OPH activity in a saline environment, sIHSELM and purified OPH were incubated under different concentrations of NaCl solutions (20 g L^{-1}, 30 g L^{-1}, 50 g

L^{-1}) at 25 °C for 4 days. The OPH activity was evaluated and calculated as described aforementioned.

1.20 The genetic stability of the engineered strains

BL21-pBV220-φX174E/pACYCDuet-SPT_3-mCherry strain was cultured with LB medium in an oscillating incubator at 37 °C overnight, with the medium being supplemented with both chloramphenicol and ampicillin. Subsequently, 10 μL of the overnight culture was inoculated into 3 mL of fresh LB medium under three different conditions, with the process repeated every 12 h: (1) LB medium supplemented with both chloramphenicol and ampicillin, (2) LB medium supplemented without any antibiotic, (3) LB medium without any antibiotic but supplemented with IPTG (0.1 mM). At predetermined time, bacterial samples were collected, diluted at various gradients, and enumerated using the plate count method. The enumeration was performed on LB agar plates supplemented with no antibiotic, with chloramphenicol alone, or with ampicillin alone, respectively. The plasmid retention rate was calculated as the ratio of CFU of bacteria on plates containing with antibiotic (either chloramphenicol or ampicillin) to those on plates without any antibiotic.

1.21 Statistical analysis

The data were presented as the mean ± standard deviation (SD). When the two groups were compared, Student's *t*-test was performed. Differences among multiple groups were evaluated using the one-way ANOVA test. All data were analyzed by GraphPad Prism (8.3.0) and ImageJ 2.1.0. GraphPad Prism (8.3.0) was also applied for all statistical analyses. Statistical significance was expressed as $*P < 0.05$, $**P < 0.01$, $***P < 0.001$, $****P < 0.0001$.

2. Supplementary Figures

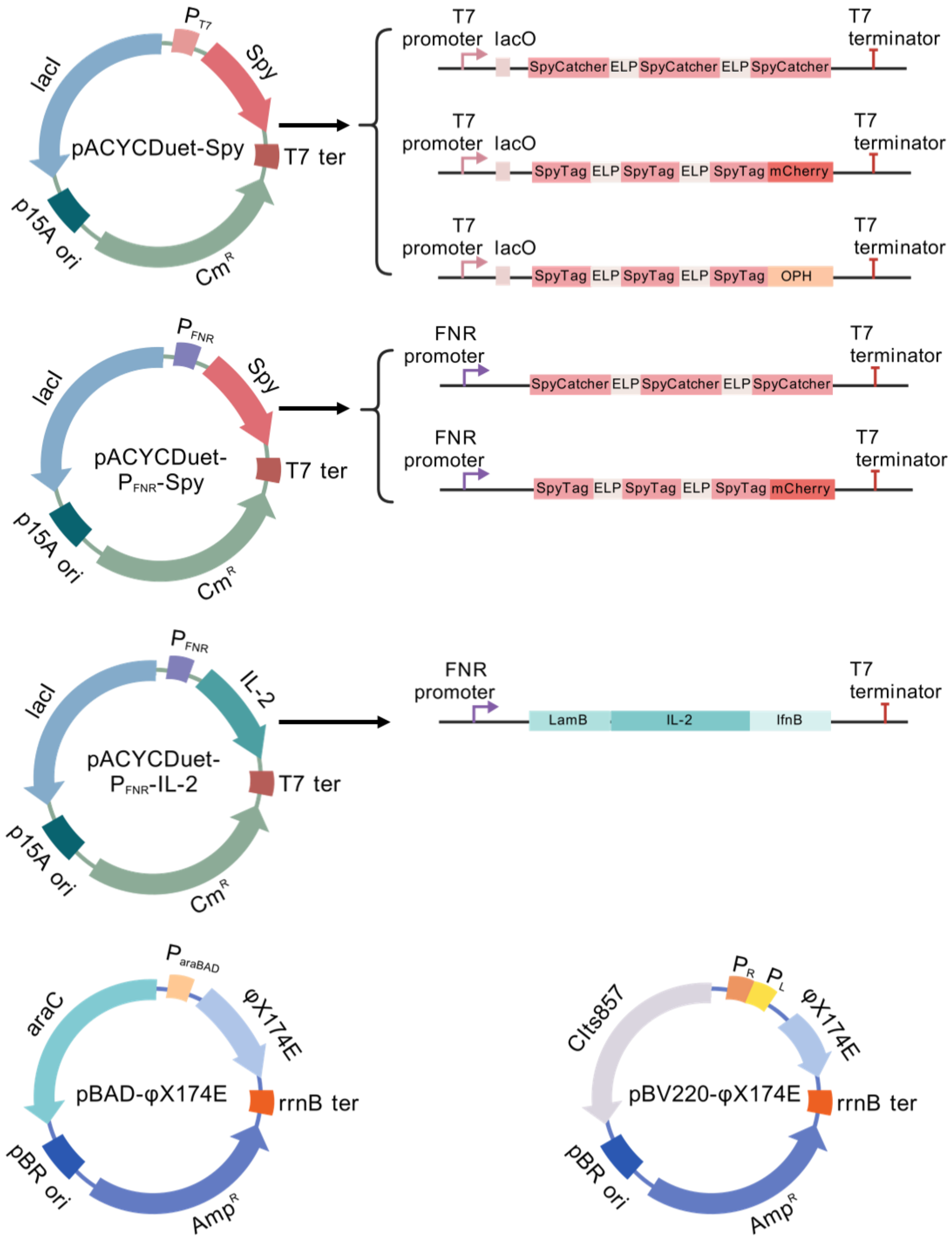

Figure S1. The main plasmids used in this study.

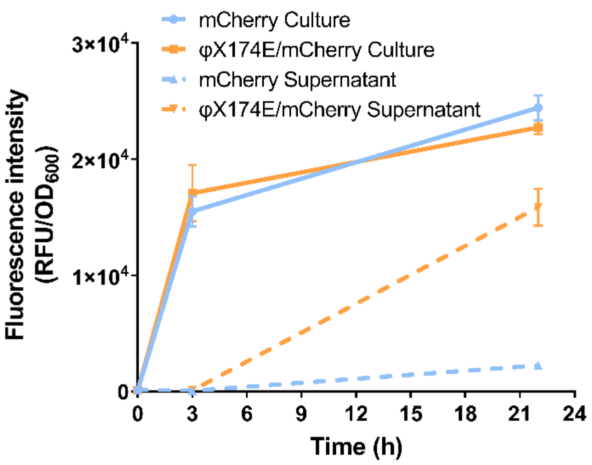

Figure S2. Fluorescence intensity of mCherry released from the culture medium or supernatant medium of the BL21(DE3) expressed with E protein (φX174E/ mCherry) or without E protein (mCherry) (n = 3). Data are presented as mean values ± SD.

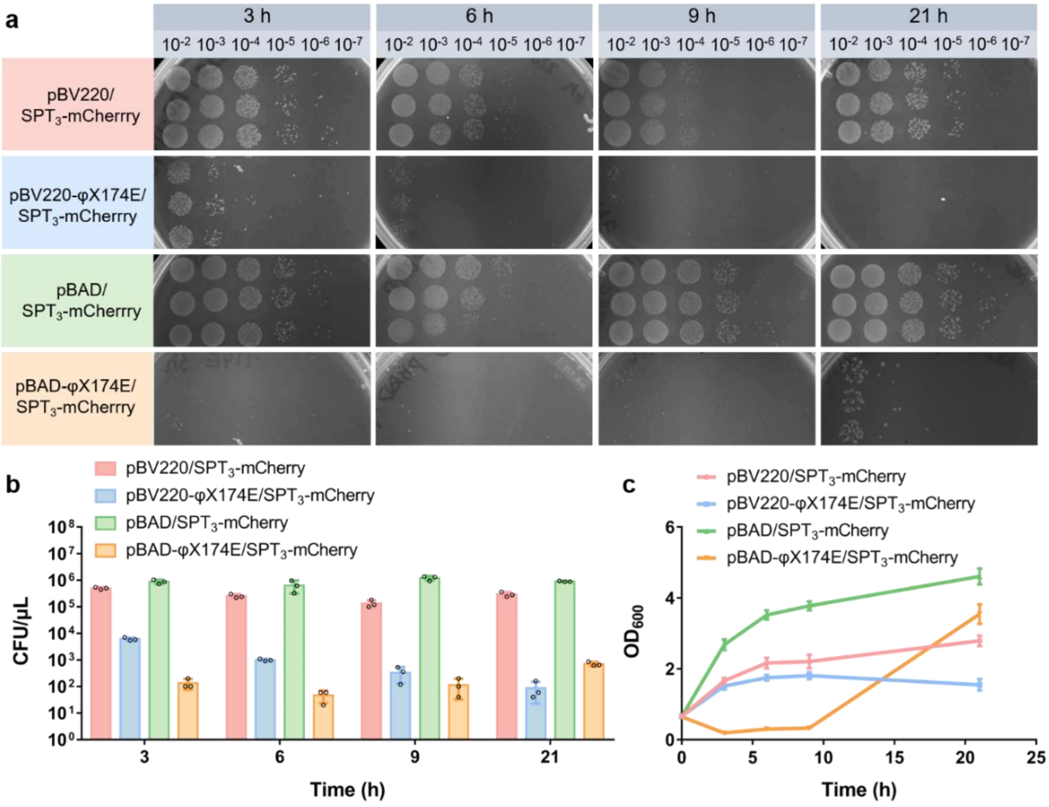

Figure S3. The lysis efficiency induced with temperature or arabinose. The changes in CFU (a, b) and $OD_{600\ nm}$ (c) of BL21(DE3) bacteria harboring either φX174E gene or no such gene within different vectors (pBV220 or pBAD). Data are presented as mean values ± SD (n = 3).

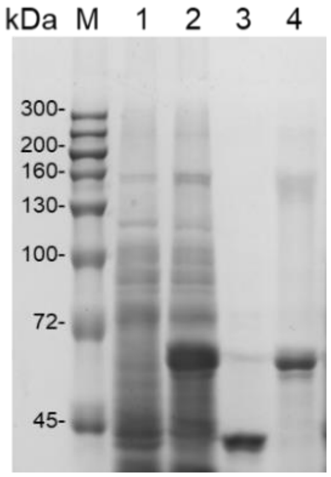

Figure S4. SDS-PAGE analysis of the expression and purification of SPC_3 and SPT_3-mCherry proteins. M, maker. 1, Supernatant of disrupted bacteria expressing SPT_3-mCherry. 2, Supernatant of disrupted bacteria expressing SPC_3. 3, Purified SPT_3-mCherry protein. 4, Purified SPC_3 protein.

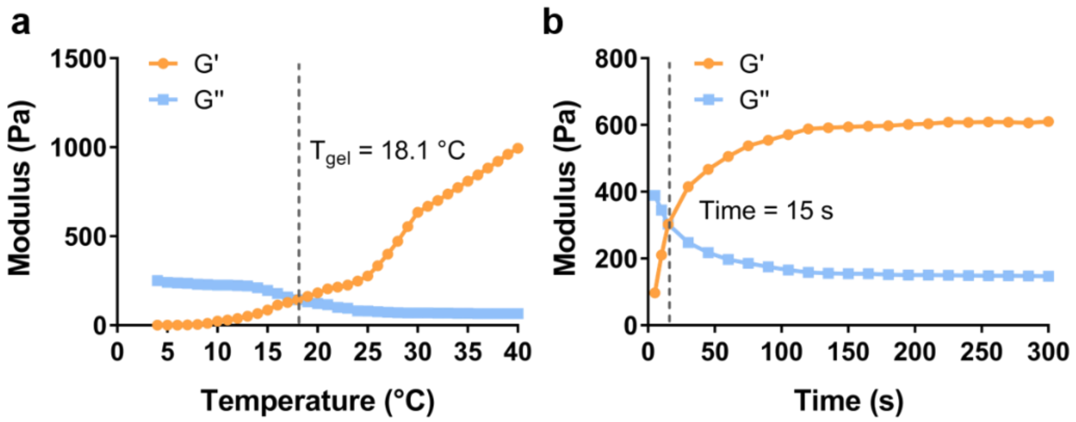

Figure S5. Rheological analysis of HBC hydrogels. (a) Temperature dependence of storage modulus G′ and loss modulus G″ of 5 wt% HBC aqueous solution. (b) Time dependence of G′ and G″ of 5 wt% HBC aqueous solution at 37 °C.

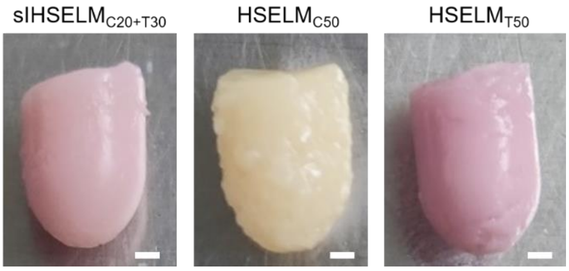

Figure S6. The representative photos of sIHSELM$_{C20+T30}$, HSELM$_{C50}$ and HSELM$_{T50}$. The scale bar is 2 mm.

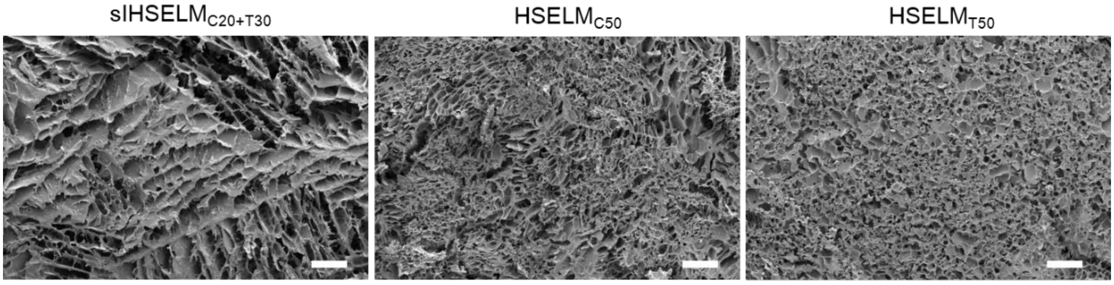

Figure S7. SEM images of sIHSELM$_{C20+T30}$, HSELM$_{C50}$, and HSELM$_{T50}$. The scale bar is 100 μm.

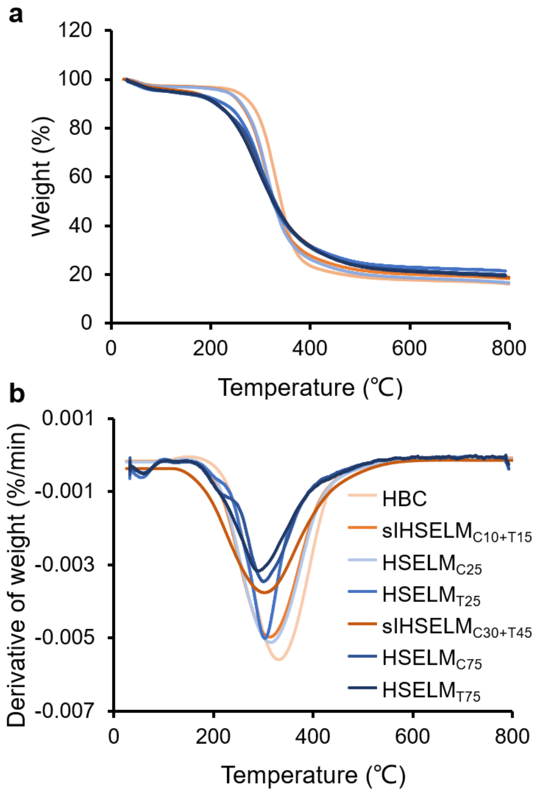

Figure S8. TGA (a) and DTG (b) profiles of HBC, sIHSELM$_{C10+T15}$, sIHSELM$_{C20+T30}$, sIHSELM$_{C30+T45}$, HSELM$_{C25}$, HSELM$_{T25}$, HSELM$_{C50}$, HSELM$_{T50}$, HSELM$_{C75}$ and HSELM$_{T75}$ samples.

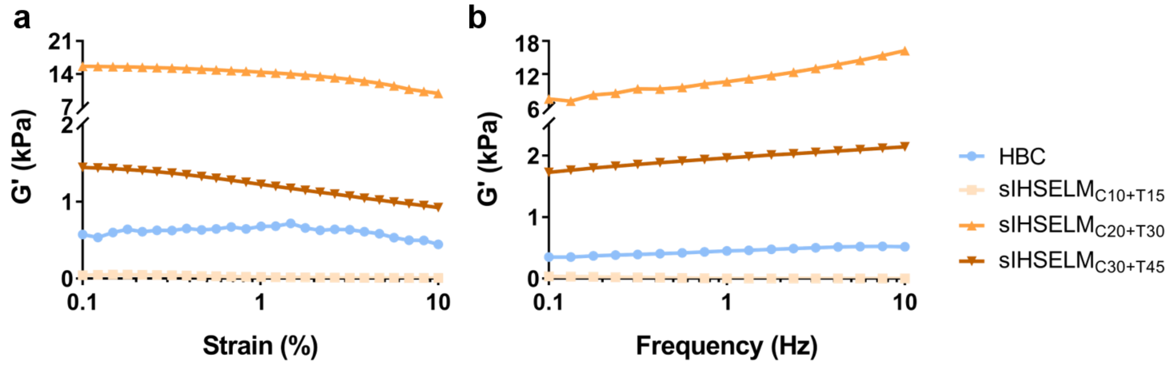

Figure S9. Rheological analysis of HBC and different sIHSELM hydrogels. (a) Strain sweep with a range of 10% to 0.1% at a constant frequency of 1 Hz. (b) Frequency sweep with a range of 0.1 Hz to 10 Hz at a constant strain of 1%.

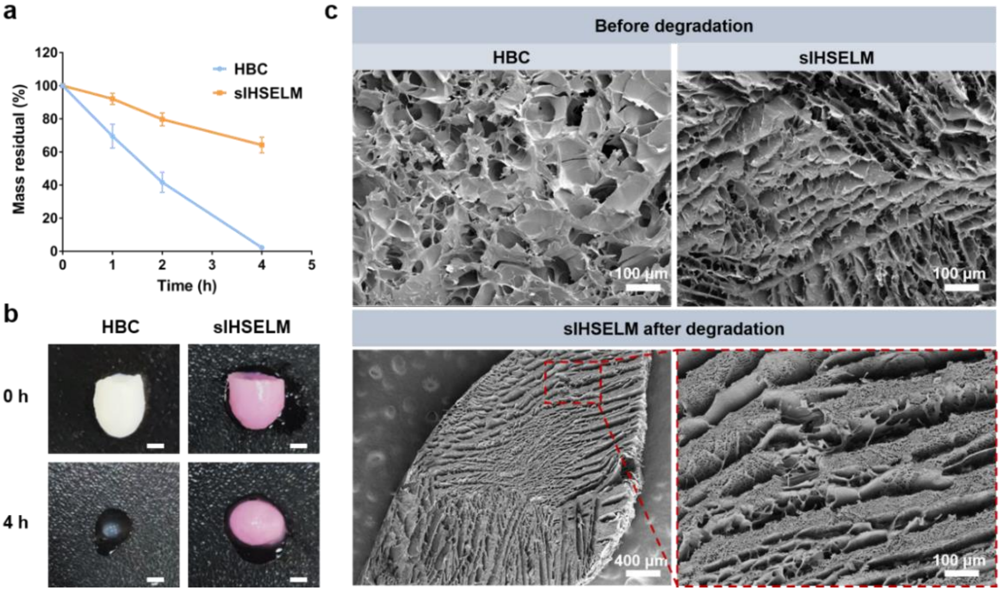

Figure S10. Chitosanase degradation analysis. (a) The mass loss profiles of HBC and sIHSELM hydrogels (n = 3). (b) The photos of HBC and sIHSELM hydrogels before and after incubated with chitosanase for 4 h. The scale bar is 2 mm. (c**)** The microstructures of HBC and sIHSELM hydrogels before and after degradation observed by SEM. The scale bar is 100 μm and 400 μm, respectively. Data are presented as mean values ± SD.

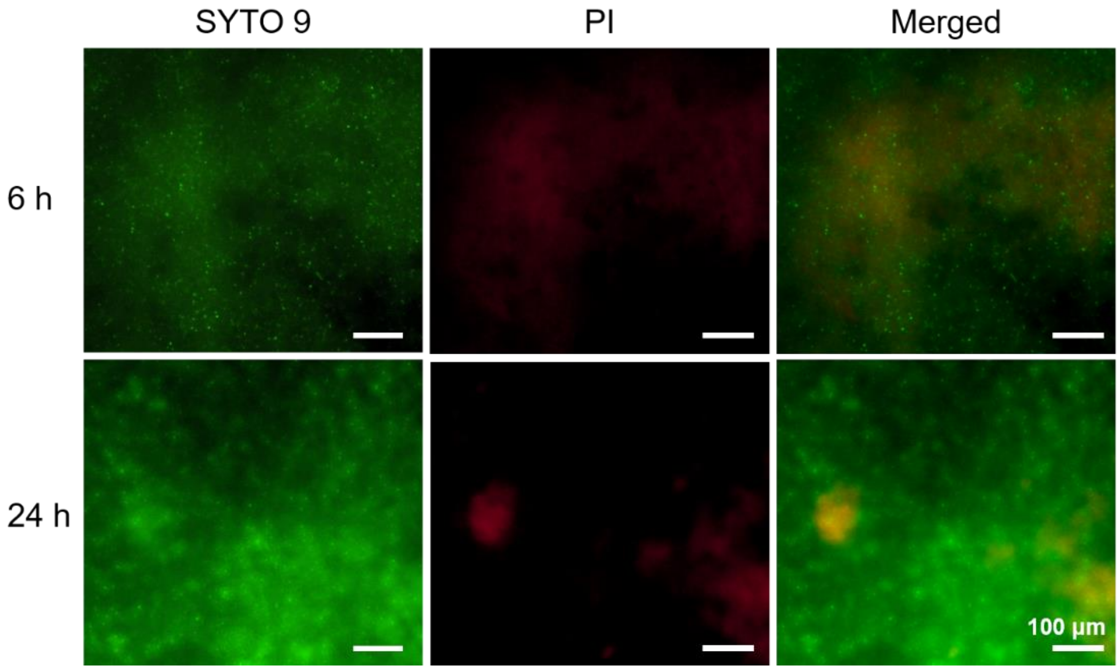

Figure S11. The observation of live (stained with SYTO 9) and dead (stained with PI) bacteria within sIHSELM over a period of 24 h. The scale bar is 100 μm.

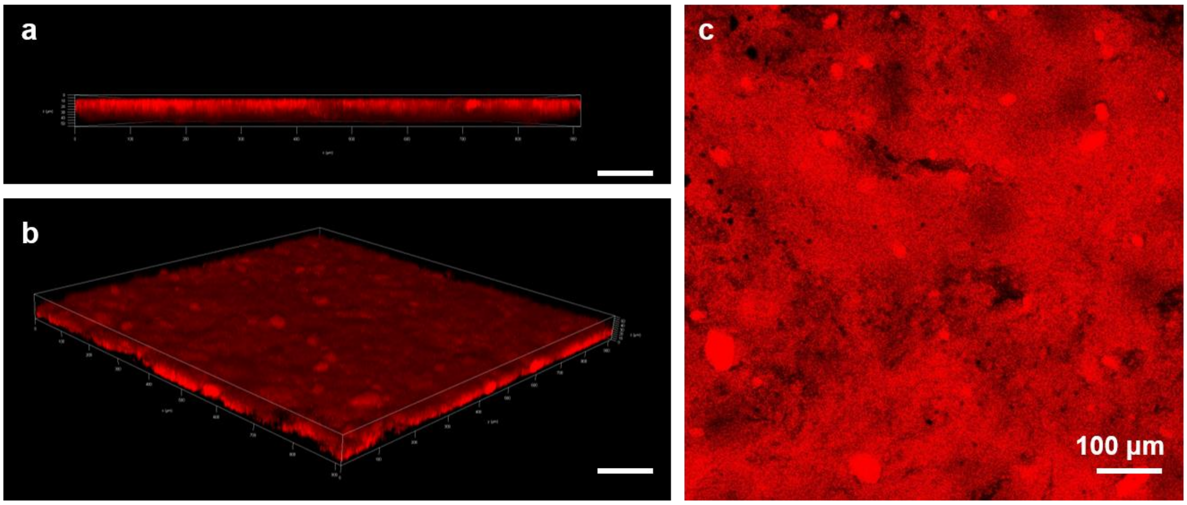

Figure S12. Spatial distribution of SPT_3-mCherry protein within the sIHSELM hydrogel. 3D (a, b) and z-axis maximum projection (c) views of CLSM images. The scale bar is 100 μm.

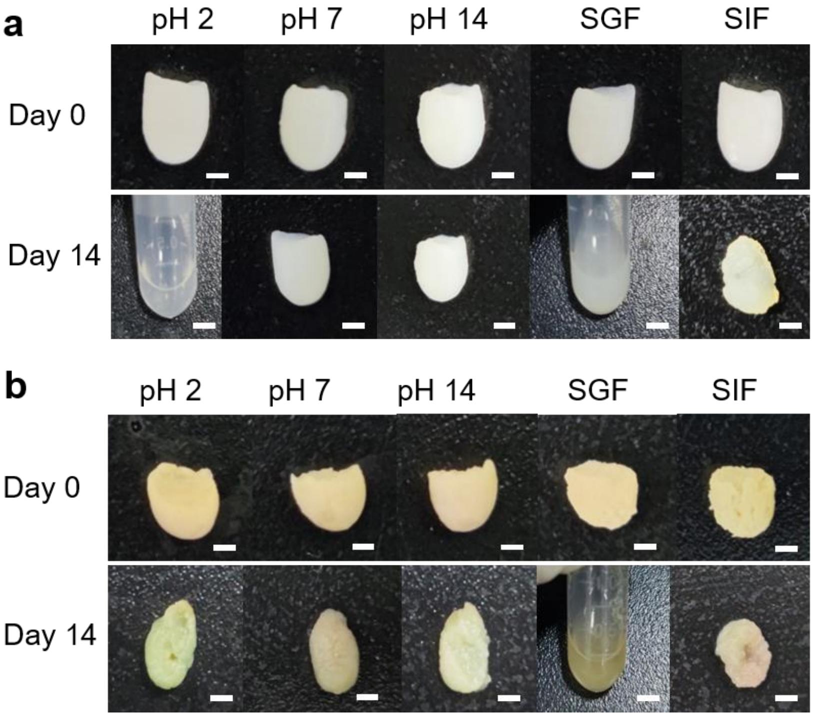

Figure S13. The representative photos of HBC (a) and HSELM (b) before and after incubated in different conditions. The scale bar is 2 mm.

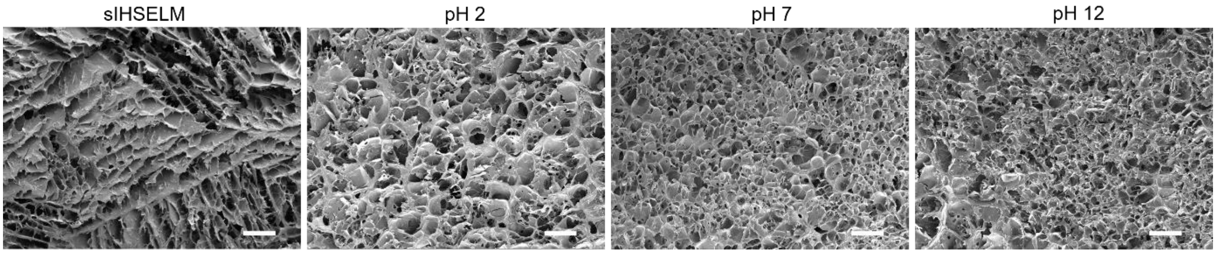

Figure S14. SEM images of sIHSELM hydrogels before incubation and subsequent to incubation in pH 2, pH 7 and pH 12 solutions for 14 days. The scale bar is 100 μm.

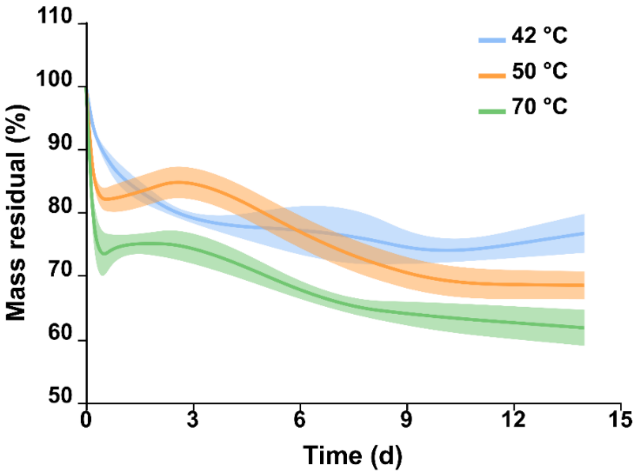

Figure S15. The stability of sIHSELM under different temperatures ($n = 3$). Data are presented as mean values ± SD.

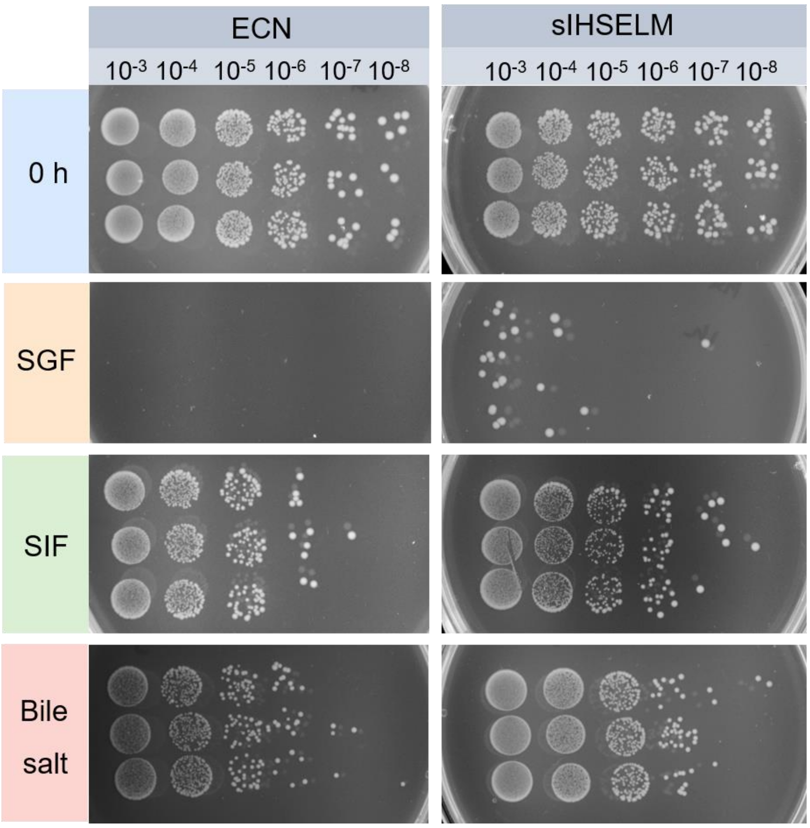

Figure S16. Tolerance of ECN encapsulated within or without sIHSELM after treatment with SGF, SIF and bile salt for 2 h (n = 3).

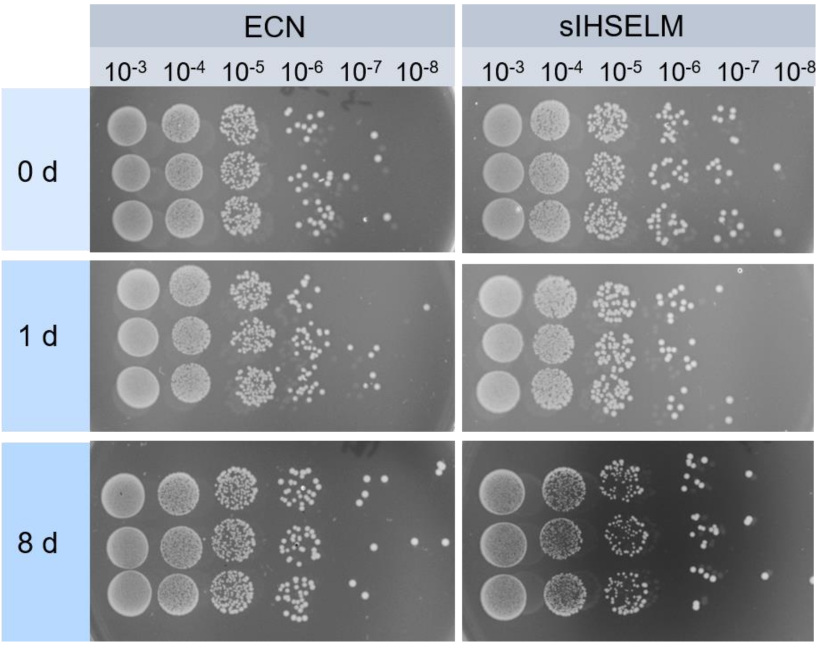

Figure S17. The survival of ECN encapsulated within or without sIHSELM when stored at -80 °C for a duration of 8 days (n = 3).

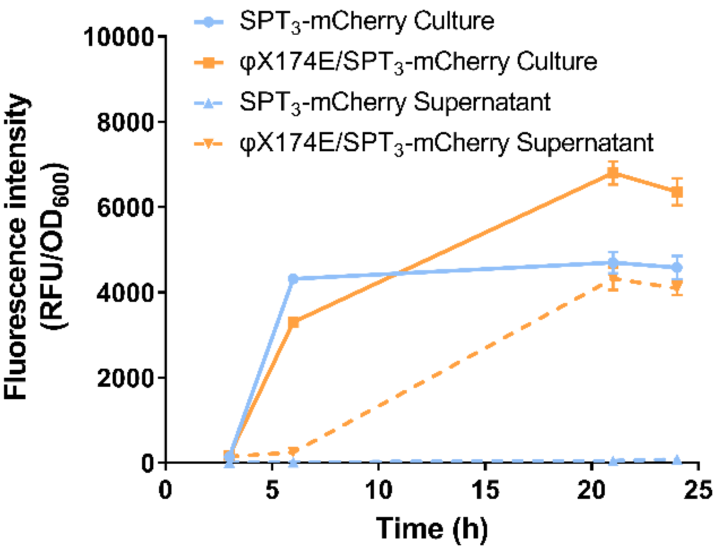

Figure S18. Fluorescence intensity of mCherry released from the culture medium or supernatant medium of the ECN expressed with E protein (φX174E/mCherry) or without E protein (mCherry) and induced by arabinose. Data are presented as mean values ± SD (n = 3).

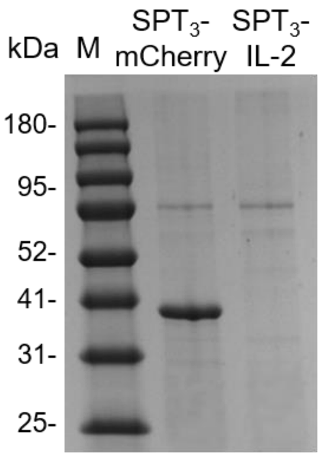

Figure S19. SDS-PAGE analysis of the purified SPT_3-mCherry and SPT_3-IL-2 proteins.

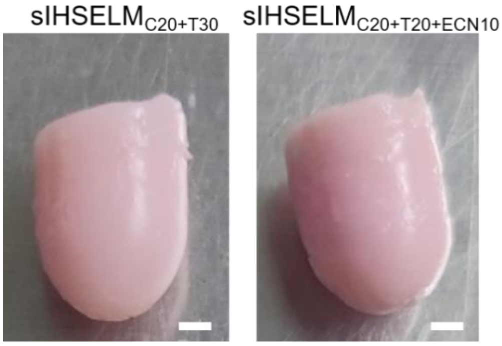

Figure S20. The representative photos of sIHSELM$_{C20+T30}$ and sIHSELM$_{C20+T20+ECN10}$. The formulation of sIHSELM$_{C20+T20+ECN10}$ was utilized for IBD treatment. The scale bar is 2 mm.

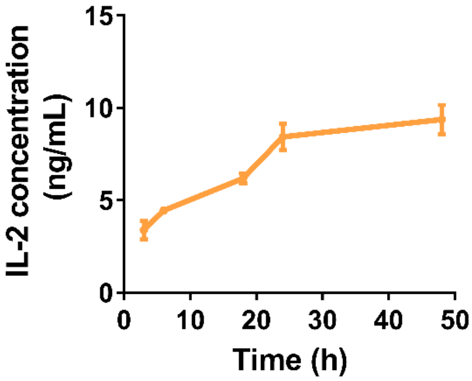

Figure S21. The *in vitro* release profiles of IL-2 from sIHSELM. Data are presented as mean values ± SD (n = 3).

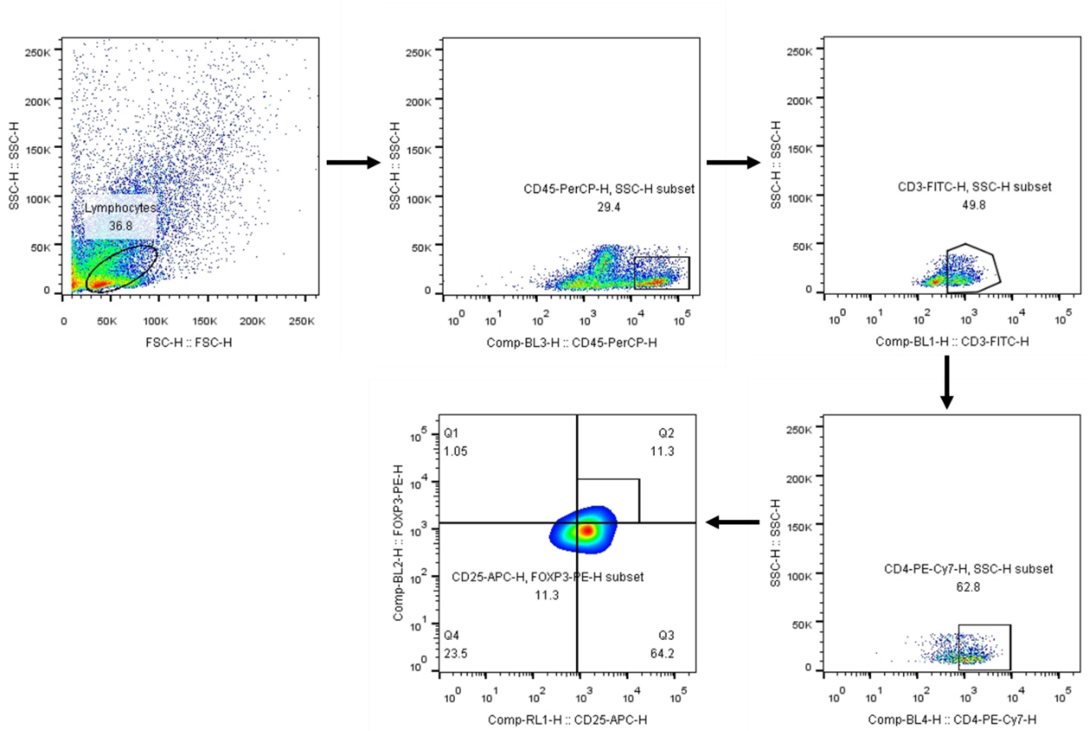

Figure S22. Representative gating strategy used for flow cytometry analysis of Foxp3$^+$CD4$^+$ cells in colon tissues of mice with different treatments.

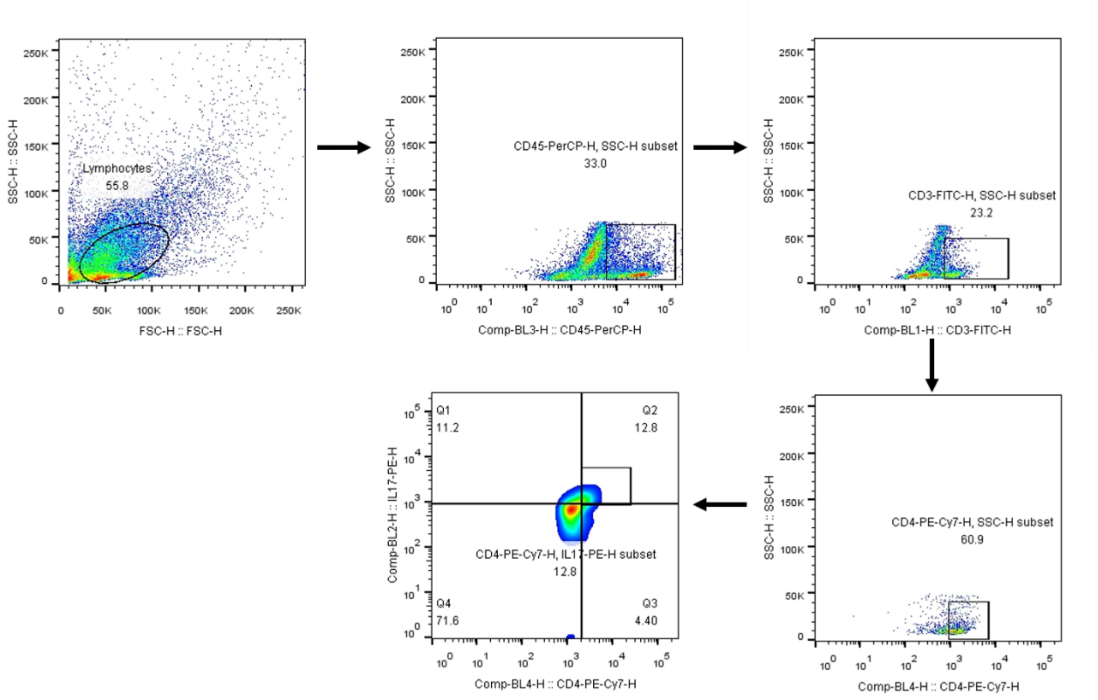

Figure S23. Representative gating strategy used for flow cytometry analysis of IL-17a$^+$CD3$^+$ cells in colon tissues of mice with different treatments.

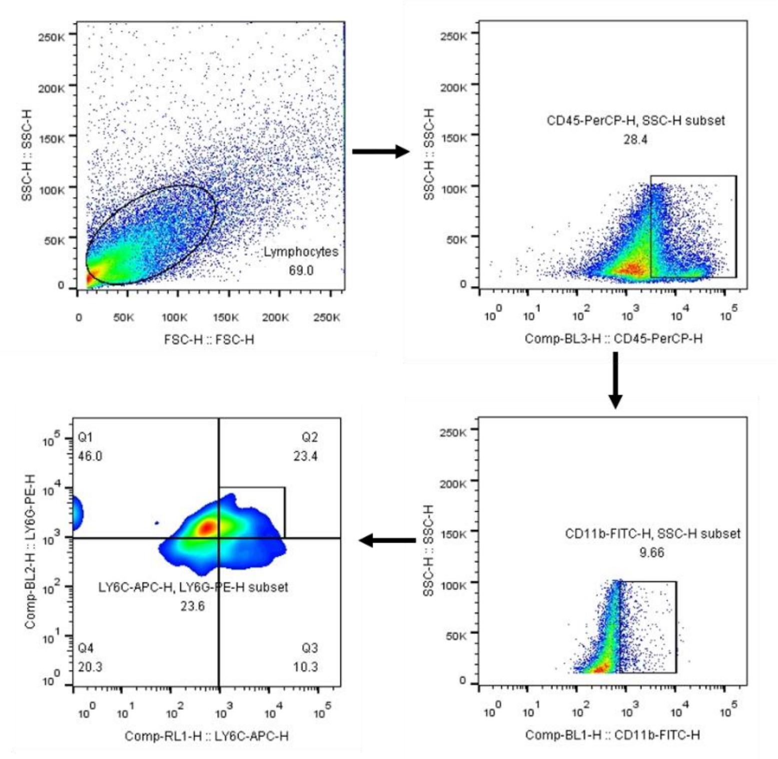

Figure S24. Representative gating strategy used for flow cytometry analysis of Ly6G$^+$CD45$^+$CD11b$^+$ cells in colon tissues of mice with different treatments.

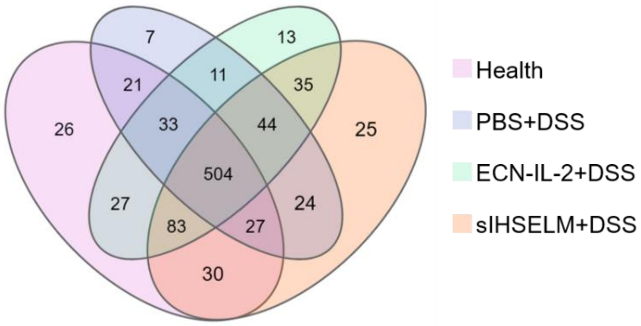

Figure S25. Venn diagram of the bacterial species in the colons harvested from the mice with different treatments.

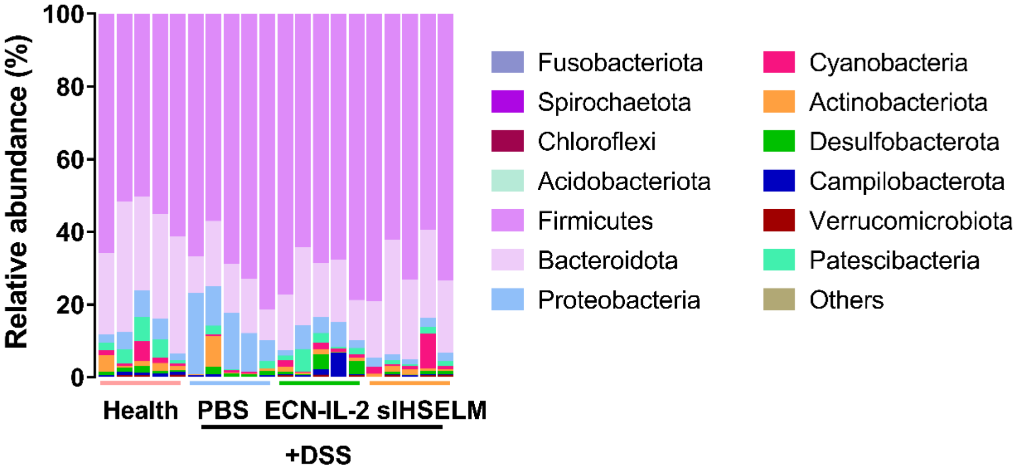

Figure S26. Relative abundance of different phylums in the gut microbiota of mice with different treatments.

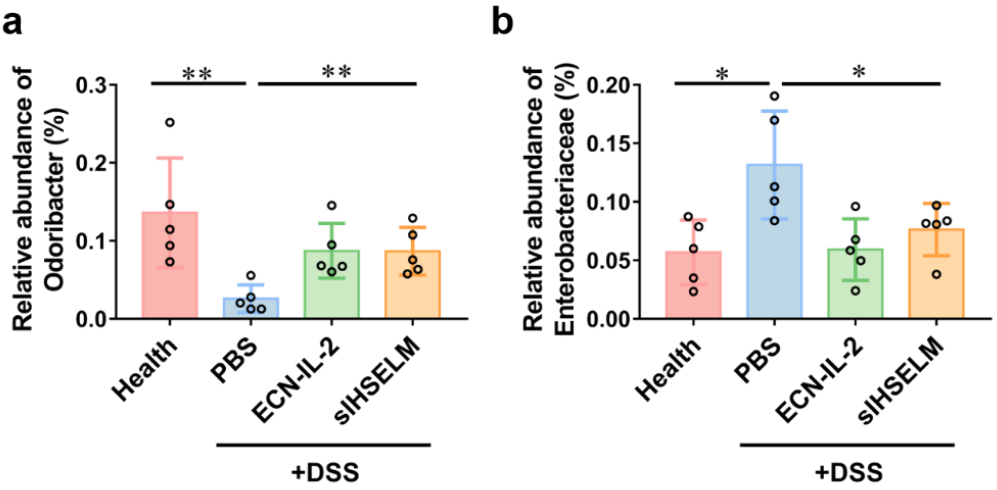

Figure S27. Relative abundances of Odoribacter (a) and Enterobacteriaceae (b) (n = 3). Data are presented as mean values ± SD.

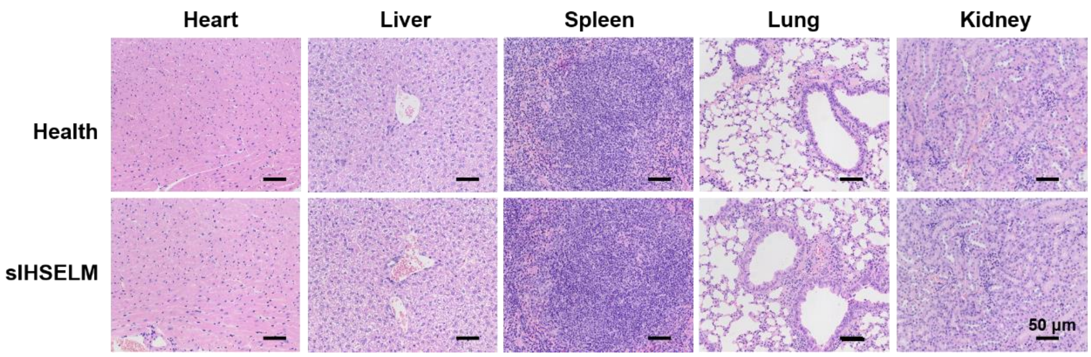

Figure S28. H&E staining images of heart, liver, spleen, lung and kidney collected from mice with different treatments on day 11. The scale bar is 50 μm.

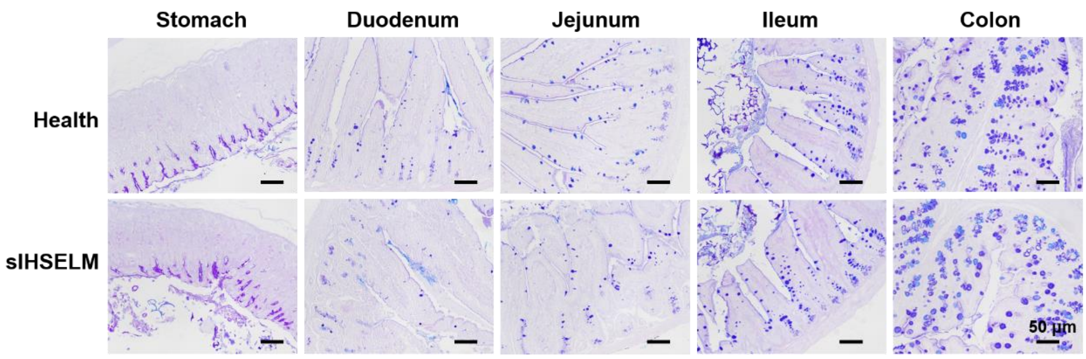

Figure S29. AB-PAS staining images of stomach, duodenum, jejunum, ileum and colon collected from mice with different treatments on day 11. The scale bar is 50 μm.

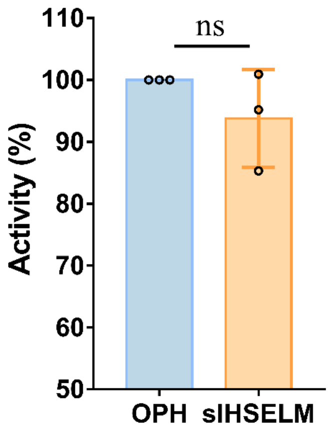

Figure S30. The relative enzyme activity of OPH encapsulated within or without sIHSELM (n = 3). Data are presented as mean values ± SD.

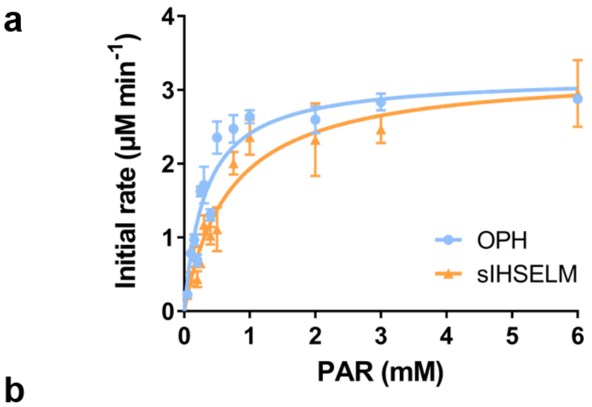

b

Sample	k_{cat} (s^{-1})	K_m (mM)	k_{cat}/K_m (mM^{-1} s^{-1})
OPH	4.1 ± 0.2	0.32 ± 0.04	12.7 ± 1.6
sIHSELM	4.2 ± 0.2	0.69 ± 0.10	6.1 ± 0.9

Figure S31. The enzyme kinetics of OPH and sIHSELM. (a) Michaelis-Menten curves. (b) Averaged enzyme activities listed in the tables. Data are presented as mean values ± SD (*n* = 3).

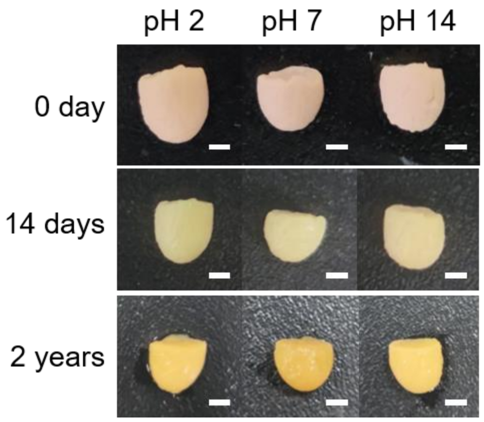

Figure S32. The photos of sIHSELM before and after incubated in different conditions for two years. The scale bar is 2 mm.

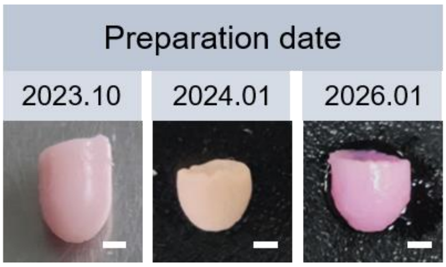

Figure S33. The photos of sIHSELM prepared in different batches. The scale bar is 2 mm.

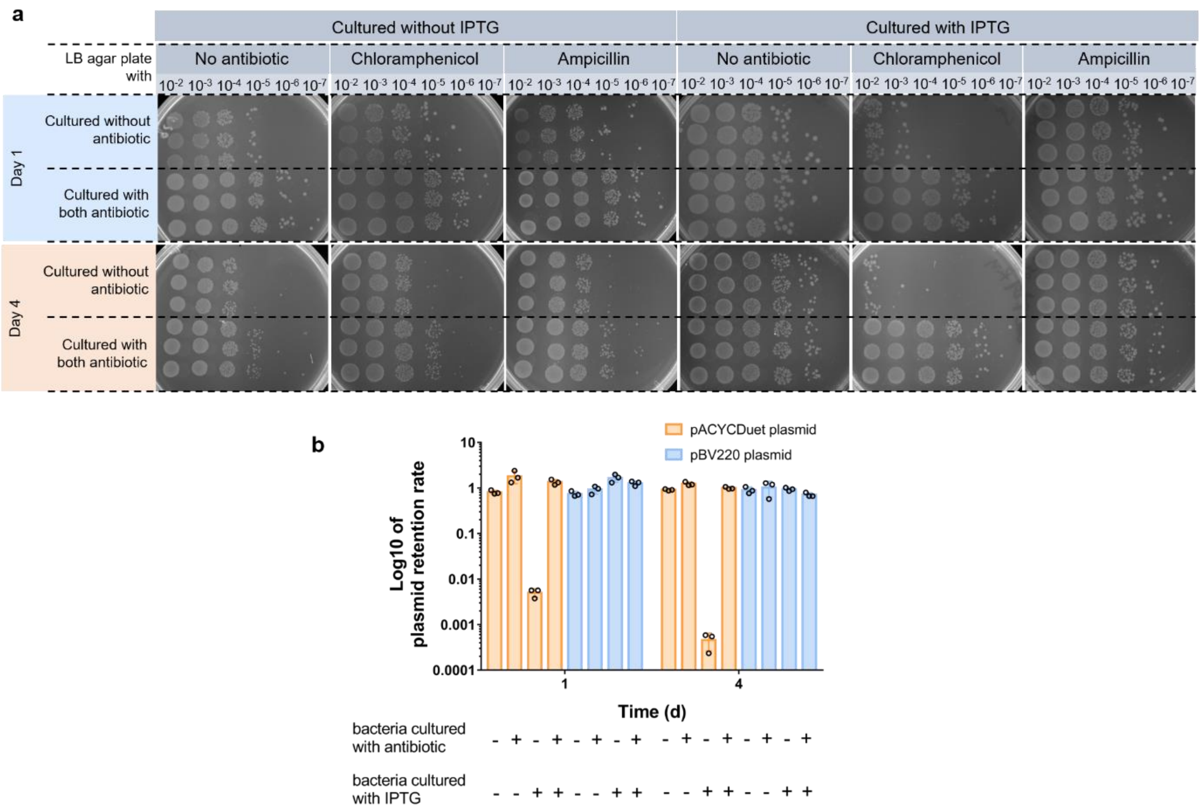

Figure S34. The plasmid retention profiles. (a) Photos of LB agar plates for CFU count. (b) The plasmid retention rates. The pACYCDuet plasmid exhibited instability in the presence of an inducer (e.g., IPTG) within the culture medium without antibiotic, as the expression of induced protein imposed growth and metabolic stress on the host cells. Conversely, owing to the tightly regulated T7 promoter, the pACYCDuet plasmid maintained stability under conditions without inducer supplementation. Regarding the pBV220 plasmid, due to the stringent repression mechanism of the P_L/P_R promoter system, protein leakage expression was minimal, thereby ensuring its stable retention within bacterial cells at 37 °C. Data are presented as mean values ± SD (n = 3).

3. Supplementary Tables

Table S1. The maximum thermal decomposition temperature (T_{max}) of different HBC, sIHSELM and HSELM samples.

Sample	T_{max} (°C)
HBC	330.7
sIHSELM$_{C10+T15}$	312
HSELM$_{C25}$	314.5
HSELM$_{T25}$	303.0
sIHSELM$_{C20+T30}$	308.2
HSELM$_{C50}$	295.7
HSELM$_{T50}$	300.5
sIHSELM$_{C30+T45}$	302.2
HSELM$_{C75}$	300.2
HSELM$_{T75}$	289.9

Table S2. The criteria for scoring disease activity index (DAI).

Score	Weight loss (%)	Stool consistency	Blood in stool
0	None	Normal	Normal
1	1-5	Slight loose stool	Small presence of blood
2	5-10	Loose stool	Significant presence of blood
3	10-15	Diarrhea	Gross blood
4	> 15		

Table S3. Histological grading scheme for DSS colitis.

Colonic epithelial damage		Inflammatory cell infiltration	
		Mucosa	
Score	Description	Score	Description
0	Normal	0	Normal

1	Hyperproliferation, irregular crypts, and goblet cell loss	1	Mild
		2	Modest
2	Mild to moderate crypt loss (10 -50%)	3	Severe
		Submucosa	
3	Severe crypt loss (50-90%)	Score	Description
		0	Normal
4	Complete crypt loss, surface epithelium intact	1	Mild to modest
		2	Severe
5	Small-to medium-sized ulcers (< 10 crypt widths)	Muscle/serosa	
		Score	Description
6	Large ulcers (≥10 crypt widths)	0	Normal
		1	Moderate to severe

Table S4. The gene sequences used in this study.

Genes	Sequences
SpyCatcher-	gcgatggtggataccctgagcggtttaagcagcgaacagggccaaagcggtgatatgacca
ELP-	ttgaagaagatagcgcgacccatattaaatttagcaaacgcgatgaagatggcaaggagctg
SpyCatcher-	gcgggcgcaacgatggaattacgtgatagcagcggtaaaaccattagcacctggattagcg
ELP-	atggccaggtgaaagatttttatctgtatccgggcaaatacacctttgtggaaaccgcggcgc
SpyCatcher	cggatggctatgaagttgcgaccgcgattacctttaccgtgaatgaacagggccaggtgacc
	gtgaatggcaaagcgaccaaaggcgatgcgcatattctcgacgtaggcgtgccgggcgtg
	ggtgtaccgggcggtggcgttccgggcgctggcgttcctggcgttggtgttccgggtgttgg
	cgtgccgggtgtgggtgtccctggtggtggcgttccgggcgcgggtgtgccgggcggcgg
	tgttccgggtgttggcgtcccaggtgttggtgtgccgggcggtggtgttcctggcgcgggtgt
	gccaggcgttggtgtgccgggtgagctcgcgatggtggataccctgagcggtttaagcagc
	gaacagggccaaagcggtgatatgaccattgaagaagatagcgcgacccatattaaatttag
	caaacgcgatgaagatggcaaggagctggcgggcgcaacgatggaattacgtgatagcag
	cggtaaaaccattagcacctggattagcgatggccaggtgaaagatttttatctgtatccgggc
	aaatacacctttgtggaaaccgcggcgccggatggctatgaagttgcgaccgcgattacctttt

	accgtgaatgaacagggccaggtgaccgtgaatggcaaagcgaccaaaggcgatgcgcat<br>attactagtgttggcgtgccgggcgtgggtgttccgggcggcggtgttcctggcgcgggcgt<br>tccgggtgtgggtgttccgggcgttggcgttccaggtgtgggtgtgccaggcggcggcgtg<br>ccgggtgcgggtgttccaggtggcggcgttccaggcgttggcgttccaggtgtgggcgttc<br>ctggcggcggcgttccgggtgcgggtgtaccgggcggcggcgtaccgggtctcgacgcg<br>atggtggataccctgagcggtttaagcagcgaacagggccaaagcggtgatatgaccattg<br>aagaagatagcgcgacccatattaaatttagcaaacgcgatgaagatggcaaggagctggc<br>gggcgcaacgatggaattacgtgatagcagcggtaaaaccattagcacctggattagcgatg<br>gccaggtgaaagatttttatctgtatccgggcaaatacacctttgtggaaaccgcggcgccgg<br>atggctatgaagttgcgaccgcgattacctttaccgtgaatgaacagggccaggtgaccgtg<br>aatggcaaagcgaccaaaggcgatgcgcatatt
SpyTag-ELP-SpyTag-ELP-SpyTag-mCherry	gcgcatattgttatggttgatgcgtataaaccgaccaaactcgacggtcatggtgttggtgttcc<br>gggtgttggtgttccgggtgttggtgttccgggtgttggtgagctcgcgcatattgttatggttg<br>atgcgtataaaccgaccaaaactagtgttccgggtgttggtgttccgggtgttggtgttccggg<br>tgaaggtggtttactcgacgcgcatattgttatggttgatgcgtataaaccgaccaaactcgag<br>ggcggtggtggtagcatggtgagcaagggcgaggaggataacatggccatcatcaaggag<br>ttcatgcgcttcaaggtgcacatggagggctccgtgaacggccacgagttcgagatcgagg<br>gcgagggcgagggccgcccctacgagggcacccagaccgccaagctgaaggtgaccaa<br>gggtggcccccctgcccttcgcctgggacatcctgtcccctcagttcatgtacggctccaaggc<br>ctacgtgaagcaccccgccgacatccccgactacttgaagctgtccttccccgagggcttca<br>agtgggagcgcgtgatgaacttcgaggacggcggcgtggtgaccgtgacccaggactcct<br>ccctgcaggacggcgaattcatctacaaggtgaagctgcgcggcaccaacttcccctccga<br>cggccccgtaatgcagaagaagaccatgggctgggaggcctcctccgagcggatgtaccc<br>cgaggacggcgccctgaagggcgagatcaagcagaggctgaagctgaaggacggcggc<br>cactacgacgctgaggtcaagaccacctacaaggccaagaagcccgtgcagctgcccggc<br>gcctacaacgtcaacatcaagttggacatcacctcccacaacgaggactacaccatcgtgga<br>acagtacgaacgcgccgagggccgccactccaccggcggcatggacgagctgtacaag
φX174E	atggttcgttggaccctgtgggataccctggcgttcctgctgctgctgtctctgctgctgccga<br>gcctgctgatcatgttcattccgtctacctttaaacgtccggtttcttcttggaaagctctgaacct<br>gcgtaaaactctgctgatggcttcttctgttcgtctgaaaccgctgaactgttctcgtctgccgtg

	cgtttatgctcaggaaaccctgaccttcctgctgacccagaagaaaacctgcgttaaaaacta<br>cgttcgtaaagaataa
OPH	atgtctatcggtactggtgaccgtatcaacaccgttcgtggtccgatcaccatctctgaagcgg<br>gtttcaccttgactcacgaacacatctgcggttcttctgctggctttctgcgcgcatggccgga<br>gttcttcggttctcgtaaagctctggctgagaaagcggttcgtggtttgcgtcgtgctcgtgctg<br>cgggtgttcgtaccatcgttgacgtttctaccttcgacatcggtcgtgatgtttctctgctggcag<br>aagtaagccgtgcagcagacgttcacatcgttgctgcgaccggtctgtggttcgatccaccg<br>ctgtctatgcgtctgcgttctgttgaagaactgactcagttctttctgcgtgaaatccagtacggt<br>atcgaggacaccggtatccgtgcaggtatcatcaaagttgcgaccaccggtaaagcgactcc<br>gttccaagaactggttctgaaagcagcagctcgtgcgtctctggctactggtgttccggttacc<br>actcacaccgcagcatctcagcgtgatggtgaacagcaggcggcaatcttcgaatctgaag<br>gtctgtctccgagccgtgtttgcatcggtcattctgacgacactgacgatctgtcttacctgact<br>gcgctggctgctcgtggttacctgatcggcctggatcacatcccgcactctgcgatcggtctg<br>gaagataacgcttctgcgtctgctttgctgggtatccgtagctggcagactcgtgcgctgctga<br>tcaaagcgctgatcgaccagggttacatgaaacaaatcttggtttccaacgactggctgttcg<br>gtttcagctcttacgttaccaacatcatggacgttatggaccgtgttaatccggacggtatggc<br>gttcattccgctgcgtgttattccgttcttgcgtgagaaaggtgttccgcaagaaaccttggcgg<br>gtatcaccgttaccaatccagcgcgtttcttgtctccgactctgcgtgcgtcttaa
LamB-IL-2-LfnB	atgatgattactctgcgtaaactgccgctggcggttgcagttgcagcaggtgttatgtctgctca<br>ggcgatggcagctccaacctcttcttctacctctagctctaccgctgaagctcagcagcagca<br>gcaacagcaacaacaacaacagcagcacctggaacagctgctgatggacctgcaagaact<br>gctgtctcgtatggagaactaccgtaacctgaaactgccacgtatgctgaccttcaaattctac<br>ctgccgaaacaggctaccgaactgaaagaccttcagtgcctggaagacgaactgggtccgc<br>tgcgtcacgttctggacctgactcagtccaaatctttccagctcgaagacgcggagaacttcat<br>ctccaacatccgtgttaccgttgttaaactgaaaggttctgacaacacctttgaatgccagttcg<br>acgacgaatctgcgaccgttgttgactttctgcgtcgttggattgcgttctgccagtctatcatct<br>ctacctctccgcagactgacgttaccatcaagacctaa

4. References

1. Z. Bao, C. Jiang, Z. Wang, et al."The Influence of Solvent Formulations on Thermosensitive

Hydroxybutyl Chitosan Hydrogel as a Potential Delivery Matrix for Cell Therapy." *Carbohydrate Polymers* 170 (2017): 80. https://doi.org/10.1016/j.carbpol.2017.04.038.

2. Z. Bao, P. Gao, G. Xia, et al."A Thermosensitive Hydroxybutyl Chitosan Hydrogel as a Potential Co-Delivery Matrix for Drugs on Keloid Inhibition." *Journal of Materials Chemistry B* 4 (2016): 3936. https://doi.org/10.1039/C6TB00378H.

3. J.M. Dang, D.D.N. Sun, Y. Shin-Ya, et al."Temperature-Responsive Hydroxybutyl Chitosan for the Culture of Mesenchymal Stem Cells and Intervertebral Disk Cells." *Biomaterials* 27 (2006): 406. https://doi.org/10.1016/j.biomaterials.2005.07.033.

4. P. Praveschotinunt, A.M. Duraj-Thatte, I. Gelfat, et al."Engineered *E. coli* Nissle 1917 for the Delivery of Matrix-Tethered Therapeutic Domains to the Gut." *Nature Communications* 10 (2019): 5580. https://doi.org/10.1038/s41467-019-13336-6.

5. J. Zhou, M. Li, Q. Chen, et al."Programmable Probiotics Modulate Inflammation and Gut Microbiota for Inflammatory Bowel Disease Treatment after Effective Oral Delivery." *Nature Communications* 13 (2022): 3432. https://doi.org/10.1038/s41467-022-31171-0.
